# Evolution of sequence, structural and functional diversity of the ubiquitous DNA/RNA-binding Alba domain

**DOI:** 10.1038/s41598-024-79937-4

**Published:** 2024-12-05

**Authors:** Jaiganesh Jagadeesh, Shruthi Sridhar Vembar

**Affiliations:** Institute of Bioinformatics and Applied Biotechnology, Bengaluru, Karnataka India

**Keywords:** Functional clustering, Genome informatics, Phylogeny, Protein analysis, Protein function predictions, Protein structure predictions, Proteome informatics

## Abstract

The DNA/RNA-binding Alba domain is prevalent across all kingdoms of life. First discovered in archaea, this protein domain has evolved from RNA- to DNA-binding, with a concomitant expansion in the range of cellular processes that it regulates. Despite its widespread presence, the full extent of its sequence, structural, and functional diversity remains unexplored. In this study, we employed iterative searches in PSI-BLAST to identify 15,161 unique Alba domain-containing proteins from the NCBI non-redundant protein database. Sequence similarity network (SSN) analysis clustered them into 13 distinct subgroups, including the archaeal Alba and eukaryotic Rpp20/Pop7 and Rpp25/Pop6 groups, as well as novel fungal and *Plasmodium*-specific Albas. Sequence and structural conservation analysis of the subgroups indicated high preservation of the dimer interface, with Alba domains from unicellular eukaryotes notably exhibiting structural deviations towards their C-terminal end. Finally, phylogenetic analysis, while supporting SSN clustering, revealed the evolutionary branchpoint at which the eukaryotic Rpp20- and Rpp25-like clades emerged from archaeal Albas, and the subsequent taxonomic lineage-based divergence within each clade. Taken together, this comprehensive analysis enhances our understanding of the evolutionary history of Alba domain-containing proteins across diverse organisms.

## Introduction

Genome organization and gene regulation are intertwined and indispensable processes in all domains of life. In eukaryotes, these processes are largely controlled by DNA-binding proteins, histones and their regulators, while in prokaryotes, several nucleoid-associated proteins (NAPs) take up this role. NAPs can bend, bridge, wrap, and polymerize along DNA, which ultimately allows them to exert a pervasive effect on gene expression. In addition, NAPs involved in sequence-independent chromatin organization are hypothesized to take on the role of transcription factors at certain genetic loci (reviewed in Ref.^1^). One such NAP found in all archaeal species is called Alba (Acetylation Lowers Binding Affinity): as the name suggests, Alba can exist in acetylated and non-acetylated states, with the former binding to DNA with decreased affinity^2^. Since their initial discovery in archaea in the 1980s^3^, Alba-domain containing proteins have been described in a variety of lower and higher eukaryotic organisms^4^, with most organisms expressing a minimum of two Alba domain-containing proteins. Moreover, with the advent of whole genome sequencing and a concomitant increase in the number of reported protein sequences, additional domains similar to Alba have been reported, leading to the classification of an “Alba-like domain superfamily” in the InterPro database (ID: IPR036882^4^, which includes Rpp20/Pop7 and Rpp25/Pop6 protein components of the RNAse P/MRP complex, Sporulation stage V, protein S (SpoVS) of bacteria, and other Alba domain-containing proteins. Despite these discoveries, the full extent of sequence, structural, and functional diversity of Alba proteins remains largely unexplored.

Phylogenetic analyses suggested that Alba originated as an RNA-binding domain in archaea^5^, and with time, evolved to bind to DNA and regulate chromatin biology^6^. The crystal structure of an archaeal Alba homodimer revealed a domain architecture of two α-helices and four β-strands arranged in β1-α1-β2-α2-β3-β4 configuration, which is similar to the ancient IF3-C fold found at the C-terminal end of RNA-binding translation initiation factors, as well as to the fold of DNAse I^5,7^. Within archaea, Alba proteins show functional diversity: in mesophilic archaea, they are thought to be transcription factors^8^, while in euryarchaeota, they are shown to bind to RNA and potentially serve as RNA-stabilising or -folding proteins or as translational regulators^9–11^. Moreover, given that many archaeal species encode divergent Alba domain-containing proteins Alba1 and Alba2, with sequence identities as little as 30%, this protein family can form both homo- and hetero- dimers, each packaging DNA and/or RNA into higher order structures in a distinct manner^12,13^. Nonetheless, Alba-nucleic acid binding is enabled through contacts between cationic amino acid residues in the Alba multimer and sugar phosphates in the DNA or RNA backbone^9,14^.

Early on, Alba domain proteins garnered interest as putative therapeutic targets in unicellular eukaryotic pathogens. For instance, the malaria-causing Apicomplexan parasite *Plasmodium falciparum* encodes for six Alba-domain containing proteins^15–17^. Of these, PfAlba1, PfAlba2, PfAlba3 and PfAlba4 bind to both DNA and RNA^15^, and an in-depth characterization of PfAlba1 showed its role in fine-tuning the timing of translation of virulence factor-encoding mRNAs during asexual blood stage development^18^. PfAlba3 has independently been implicated in regulating the expression of sub-telomeric *var* genes, which encode for virulence-associated surface antigens required for *P. falciparum* immune evasion^16^, and as an endonuclease in vitro^19^. In contrast, very little is known about PfAlba5 and PfAlba6^20^, which were discovered more recently using an *in silic*o approach and exhibit less than 15% sequence similarity to the remaining PfAlbas^17^. In addition to *Plasmodium* sp., Alba domain-containing proteins from the Discoban parasites *Trypanosoma brucei* and *Leishmania infantum* have been implicated as master regulators of proteome remodelling during stage transitions^21–23^ while Alba proteins from *Toxoplasma gondii* are involved in translational control of gene expression^24^.

In other uni- and multi-cellular eukaryotes, Alba domain-containing proteins have been divided into two major families, namely the Ribonuclease P protein subunit p20 family (Rpp20; also called Pop7 in budding yeast *Saccharomyces cerevisiae*) and RNase P protein subunit p25 family (Rpp25; also called Pop6). Rpp20/Pop7 and Rpp25/Pop6 were initially shown to interact as a heterodimer with the P3 domain of the catalytic RNA of the RNase P/MRP holoenzyme, which is an essential regulator of tRNA maturation in all domains of life^25,26^. Besides, these subunits can form homodimers in vitro, although the functional relevance of homodimerization is not known^27^. The individual subunits also function independently, for example, human Rpp20 has ATPase activity^28^ and complexes with the heat shock protein Hsp27 which is known to enhance the activity of RNase P in vitro^29^. More recently, it was shown that the binding of *S. cerevisiae* Pop6-Pop7 to the telomerase RNA as a heterodimer stabilizes the binding of other telomerase components, thus making them essential constituents of this ribonucleoprotein complex^30^. In plants, multiple Albadomain-containing proteins are present but their functions are less explored. Best studied in *Arabidopsis thaliana,* AtAlba1 and AtAlba2 are genic R-loop readers which maintain genome stability while AtAlba4 and AtAlba6 are involved in RNA metabolism, male reproductive development and heat stress response^31,32^. Stress-induced expression of Alba proteins has also been observed in *Oryza sativa*^33^. Overall, the functional diversity exhibited by Alba domain-containing proteins across different kingdoms of life hints at the evolutionary capability and the ‘innovability’ of this protein domain.

In this study, to understand the mechanisms by which the Alba domain evolved to gain divergent functions, we performed a comprehensive sequence–structure–function relationship analysis of more than 15,000 Alba domain-containing proteins from archaea, bacteria and eukaryotes. By combining sequence similarity networks (SSNs), multiple sequence alignments (MSAs), Hidden Markov Model (HMM) profiles, sequence conservation scores, de novo structural comparisons, and phylogenetic analysis, we identified thirteen distinct clusters of Alba domain-containing proteins and determined their evolutionary relatedness to each other. Intriguingly, many of these clusters are specific to certain unicellular eukaryotic lineages such as *Plasmodium, Theileria*, Saccharomycetales and other fungal groups. In addition, our analysis reclassified select Alba family members from pathogenic eukaryotes such as *Plasmodium* sp. into clades divergent from their host organism, reiterating their therapeutic potential.

## Results

### Alba proteins are found in all domains of life

To obtain a comprehensive picture of the diversity of Alba domain-containing proteins, we chose to implement iterative sequence similarity searches in PSI-BLAST^34^ against the NCBI non-redundant (nr) protein sequence database^35^, similar to a previous report^36^. To begin with, ‘seed’ sequences corresponding to well-characterised Alba domain-containing proteins described in the literature from archaea, unicellular eukaryotes, plants, fungi, and multicellular eukaryotes, and bacterial protein sequences annotated as ‘Alba-domain containing’ in the NCBI nr protein database, were used as queries in PSI-BLAST. When the resulting hits were clustered and visualized using CLANS^37^, two new clusters which did not contain any of the initial seed sequences were identified. After confirming that these clusters were truly Alba-like, representative sequences from these were used as queries in PSI-BLAST against the nr database, and clustered and visualised in CLANS, resulting in the identification of three new clusters; this process was continued until no new clusters were formed. Finally, 31 Alba domain sequences (Table 1) were used as ‘seeds’ in PSI-BLAST against the NCBI nr protein database to identify Alba proteins from all domains of life.

**Table 1:** List of Alba seed sequences used for PSI-BLAST analyses.

NCBI accession ID	Protein	Organism	Amino acid length	Alba domain range used for PSI-BLAST Analysis			PSI-BLAST Hits	References
				Start	End	Length		
Bacteria								
RZB33274^+^	Archaea-specific DNA-binding protein	*Desulfobacteraceae bacterium Eth-SRB1*	*88*	Full length			8353	NCBI Keyword Search
RKY63913^+^	DNA-binding protein Alba	*Candidatus Latescibacteria bacterium*	*93*				8615	
Archaea								
WP_069284532^+^	DNA-binding protein Alba	*Saccharolobus solfataricus*	*97*	Full length			8983	Wardleworth et al. (2002)^7^
WP_010866624^+^	DNA-binding protein Alba	*Aeropyrum pernix*	*94*				9236	Črnigoj et al. (2013)^39^
Unicellular eukaryotes								
XP_828521^+^	Hypothetical protein, conserved	*Trypanosoma brucei brucei*	*117*	Full length			8203	Mani et al. (2011)^40^, Verma et al. (2014)^33^
XP_001468603	Conserved hypothetical protein	*Leishmania infantum*	198	2	119	**117**	8764	Dupe et al. (2014)^41^
AAK81869	Unknown	*Leishmania infantum Alba3*	121	1	93	**92**	7334	
XP_002370656	Alba 2	*Toxoplasma gondii*	148	12	112	**120**	4116	Gissot et al. (2013)^24^
XP_001349383	DNA/RNA-binding protein Alba 1	*Plasmodium falciparum*	248	2	114	**112**	9961	Chêne et al. (2012)^15^, Goyal et al. (2012)^16^, Bunnik et al. (2016)^42^
XP_001350177	DNA/RNA-binding protein Alba 2	*Plasmodium falciparum*	211	9	158	**149**	8378	
XP_001347348^+^	DNA/RNA-binding protein Alba 3	*Plasmodium falciparum*	*107*	Full length			7738	
XP_001350189	DNA/RNA-binding protein Alba 4	*Plasmodium falciparum*	372	20	118	**98**	7014	
XP_001349666^+^	Conserved *Plasmodium *protein, unknown function - Alba 5	*Plasmodium falciparum*	*227*	Full length			692	Reddy et al. (2015)^17^
XP_001350437^+^	DNA/RNA-binding protein, putative - Alba 6	*Plasmodium falciparum*	*96*				46	
PVC57840.1^+^	Hypothetical protein MACJ_00001993	*Theileria orientalis*	*237*	3	229	**226**	58	PSI-BLAST Searches
Plants								
RLN35084	Chloroplast nucleoid DNA-binding protein -like protein	*Panicum miliaceum*	710	457	569	**112**	8703	Guerreiro et al. (2014)^43^
XP_015646669	Uncharacterized protein At2g34160 isoform X1	*Oryza sativa*	152	41	144	**103**	1816	PSI-BLAST Searches
Multicellular eukaryotes								
CDP95722	Bm9863	*Brugia malayi*	*163*	Full length			7281	NCBI Keyword Search
KAG1944584	Alba DNA/RNA-binding protein	*Pimephales promelas*	201	31	154	**123**	9889	
XP_024507562	DNA/RNA-binding protein Alba-like family-containing protein	*Strongyloides ratti*	186	6	131	**125**	9128	
VVC33431	DNA/RNA-binding protein Alba-like	*Cinara cedri*	178	19	143	**124**	6052	
CAP29508.1	Hypothetical protein CBG_09988	*Caenorhabditis briggsae AF16*	208	1	143	**142**	7909	
NP_005828^+^	Ribonuclease P protein subunit p20	*Homo sapiens*	*140*	Full length			6941	
NP_060263.2^+^	Ribonuclease P protein subunit p25	*Homo sapiens*	*199*				11214	
Fungi								
ATY61034	DNA RNA-binding Alba	*Cordyceps militaris*	218	49	218	**169**	5961	PSI-BLAST Searches
CUA76030	Hypothetical protein RSOLAG22IIIB_02036	*Rhizoctonia solani*	1163	1	164	**163**	5603	
KAE9410662	Hypothetical protein BT96DRAFT_778216, partial	*Gymnopus androsaceus JB14*	178	1	161	**160**	5388	
CUA78125^+^	Hypothetical protein RSOLAG22IIIB_02743	*Rhizoctonia solani*	520	221	348	**127**	6317	
KGQ85603.0^+^	Hypothetical protein MEU_04899	*Candida albicans*	171	30	169	**139**	6959	
NP_011544.1^+^	Ribonuclease P/MRP protein subunit POP6	*Saccharomyces cerevisiae*	*158*	Full length			6144	
NP_009726.3^+^	Ribonuclease P/MRP protein subunit POP7	*Saccharomyces cerevisiae*	*140*				4910	

The table lists 31 Alba domain-containing proteins shortlisted for PSI-BLAST through literature survey, keyword searches in NCBI and preliminary cluster analysis using CLANS.

^+^Indicates that the full-length protein was used for PSI-BLAST analysis.

Significant values are in bold and italics.

**Fig. 1:**
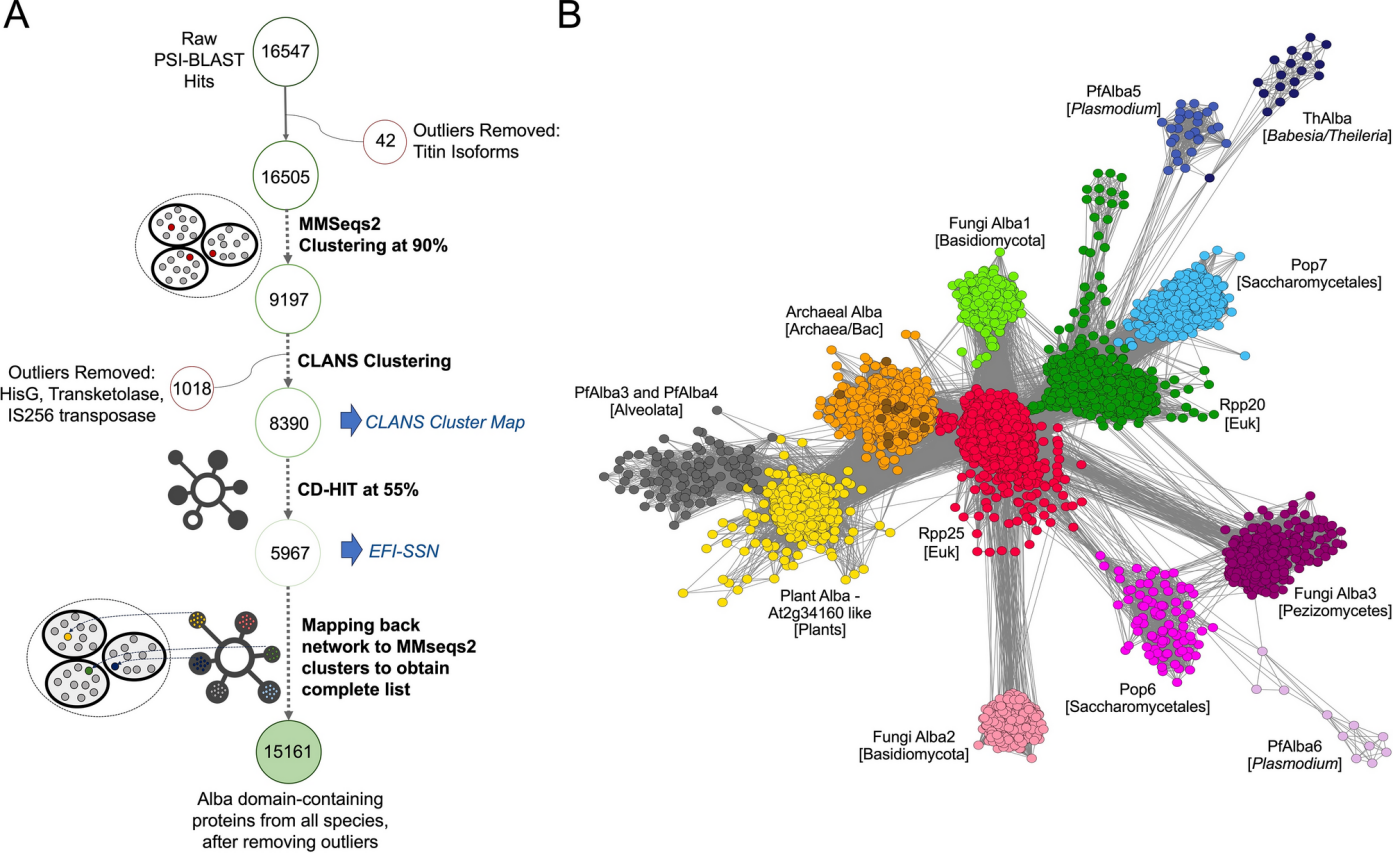
Sequence similarity network analysis reveals high diversity within Alba-like proteins. (**A**) The analysis workflow of 16,547 hits obtained by searching the NCBI protein nr database with 31 Alba seed sequences as queries in PSI-BLAST is shown schematically along with the number of sequences retained after filtering. (**B**) A representative sequence similarity network (SSN) of 15,161 proteins identified using PSI-BLAST^34^ filtered at an E-value threshold of 1E-4. Nodes represent 5967 Alba domain-containing protein sequences clustered using CD-HIT^44^ at 55% sequence identity. The SSN was generated using EFI-EST^45^ and visualized using Cytoscape^45^. Each node represents either a single protein or a representative sequence of proteins with similarity greater than 55%. The edge length represents the pairwise sequence identity between two nodes with shorter length indicative of higher sequence identity. Within the Archeal Alba cluster, bacterial nodes are highlighted in brown.

The resulting 16,547 non-redundant hits (Fig. 1A) were filtered to retain true Alba-domain containing proteins. First, 42 isoforms of the 4.2 MDa protein Titin were removed from the list since, upon closer scrutiny, they did not contain the signature Alba fold. Next, MMseqs2^38^ was used to reduce sets of closely related sequences at a maximum pairwise identity of 90% to a single representative sequence, resulting in 9197 entries. Lastly, with the help of CLANS cluster maps, outlier sequences which either remained as singletons, or clustered without forming edges with seed clusters at a pairwise BLAST p-value threshold of 1e−4, or lacked an Alba domain, were removed (Fig. 1A). The final CLANS cluster map (Supplementary Fig. S1) contained 8390 sequences which were mapped back to the original 90% MMSeqs2-reduced list, to obtain a set of 15,161 unique Alba domain-containing proteins. Each of these was checked manually for the presence of the Alba domain using HMM searches against the Alba superfamily profile (ID: IPR036882) downloaded from InterPro and compiled into a searchable Shiny R HTML file (Supplementary Material).

Taxonomic analysis of the 15,161 hits indicated that Alba domain-containing proteins are found in all domains of life (Supplementary Fig. S2), with 55 (0.4%) hits from bacteria, 3862 (25%) hits from archaea, and the remaining 11,570 (74%) hits from eukaryotes. An analysis of their amino acid length distribution (Supplementary Fig. S3) showed that the highest peak containing 1828 sequences was observed for the size range of 90–95 amino acids, which is the average size of the Alba domain in archaea. However, over 56% of the 15,161 sequences are greater than 150 amino acids in length, with the longest Alba domain-containing protein containing 4508 amino acids. Given that variations in the amino acid length of homologous proteins from different organisms can represent the diversity of functional roles that they adopt^46^, these results suggest that Alba domain-containing proteins may have diverse functions either due to sequence length variations of the Alba domain itself or due to the co-existence of other domains along with the Alba domain. A list of commonly co-occurring domains is provided in Supplementary Table S1 and includes the Thioredoxin-like_fold, TAXi_N-TAXi_C, HHH_5, TULP and RGG motifs.

### Sequence similarity network analysis reveals the diversity of Alba domain-containing proteins, particularly within unicellular eukaryotes

To obtain a global view of the primary sequence relatedness of the 15,161 Alba domain-containing proteins, a sequence similarity network (SSN) was built in the EFI-EST server^45^ using a representative set of 5967 sequences, filtered at 55% identity. An alignment score of 4, which corresponded to an e-value threshold of 1E−4, was used to determine the length of 2,033,164 edges connecting the 5967 nodes. The resulting SSN was visualised in Cytoscape^47^ using the prefuse force directed layout with 10,000 iterations. As shown in Fig. 1B, all Alba domain-containing proteins map to 13 unique clusters which correspond to archaeal Albas, plant Alba-like proteins, Rpp20-like or Rpp25-like proteins, PfAlba3/PfAlba4-like proteins, *Plasmodium* Alba5*, Plasmodium* Alba6, Alba-like proteins from *Babesia* and *Theileria* species, *Saccharomycetales* Pop6- or Pop7-like proteins, and three clusters containing fungal Alba-like proteins from Basidiomycota or Ascomycota.

Delving into the features of the SSN, we observed that the largest clusters are formed by the archaeal Albas, Rpp25-like proteins and plant Alba-like proteins (Fig. 1B; orange, red and yellow, respectively). The archaeal Alba cluster contains both archaeal and bacterial proteins, with the latter corresponding to proteins either from *Candidatus* uncultured bacteria or from metagenome assemblies (Supplementary Table S2). Connected to the archaeal Alba cluster are clusters containing Rpp25, Rpp20 or plant Alba-like proteins (Fig. 1B) suggestive of the independent evolution of these Alba homologs from the ancient archaeal Alba domain. Interestingly, the Rpp25 cluster (Fig. 1B; red) contains the largest number of sequences and occupies a pivotal position in the network, forming edges with a majority of the clusters. It contains proteins that are annotated to be Rpp25 in several uni- and multi-cellular eukaryotes, along with PfAlba1 and PfAlba2 (Supplementary Table S2). This is in keeping with published reports that have suggested that the Alba domains of PfAlba1 and PfAlba2 are homologous to Rpp25^5^. To our surprise, Pop6, which is thought to be the *S. cerevisiae* Rpp25 homolog, forms an independent cluster (Fig. 1B; pink) containing other Pop6 proteins from *Saccharomycetales* (Supplementary Table S2), with multiple edges connecting it to the Rpp25 cluster. This hints at sequence divergence between Rpp25 and *Saccharomyces* Pop6. Furthermore, three novel clusters, Fungi Alba1, Fungi Alba2 and Fungi Alba3 (Fig. 1B; fluorescent green, peach and magenta, respectively) formed edges primarily with the Rpp25 cluster and contained uncharacterised or unannotated Alba domain proteins exclusively from fungi. Two of these, Fungi Alba1 and Fungi Alba2, include proteins from Basidiomycota species (Supplementary Table S2), while the third, Fungi Alba3, contains proteins from the class Pezizomycetes within Ascomycota (Supplementary Table S2). Another cluster that intriguingly connects only with the Pop6 and Fungi Alba3 clusters is constituted by PfAlba6 and its homologs from *Plasmodium* species (Fig. 1B; mauve) suggestive of a *Plasmodium-*specific divergence/expansion of the Alba domain and that PfAlba6 may perform Pop6-like functions in *Plasmodia.*

The fourth largest cluster is formed by proteins from eumetazoa and fungal species that belong to the Rpp20 family (Fig. 1B; green) and does not contain any proteins from plants (Supplementary Table S2). It forms edges with the archaeal Alba and Rpp25 clusters, in addition to three other clusters, which are described below. In the first cluster (Fig. 1B; sky blue), we find *Saccharomycetales* proteins (Supplementary Table S2) that are similar to *S. cerevisiae* Pop7, which has been described as a homolog of Rpp20 in previous studies^5^; this independent Pop7 clustering is similar to the behaviour of the Pop6 cluster relative to Rpp25. The two other Rpp20-connected clusters are the PfAlba5 and ThAlba clusters: the former contains PfAlba5 homologs from *Plasmodium* species (Fig. 1B; blue; Supplementary Table S2) while the latter contains hypothetical proteins from *Babesia* and *Theileria* species (Fig. 1B; dark blue; Supplementary Table S2). This, again, hints at a species-specific evolution of these distant archaeal Alba relatives from Rpp20-like proteins.

Lastly, the plant Alba-like cluster (Fig. 1B; yellow) is comprised majorly of Alba domain proteins from Viridiplantae (Supplementary Table 1) and a small subset from protists such as Discoban parasites. It forms edges with the archaeal Alba cluster as well as with Rpp25 and Rpp20. In contrast, PfAlba3, PfAlba4, and their homologs from the superphylum Alveolata, cluster together (Fig. 1B; gray), and only form edges with the plant Alba-like cluster. This was surprising because previous phylogenetic studies had classified PfAlba3 and PfAlba4 as belonging to the Rpp20 family^5,48^. Indeed, given the similarity between the PfAlba5 and Rpp20 clusters in our SSN, we hypothesize that, in *P. falciparum,* the functions of Rpp20 might be carried out by PfAlba5 and not by PfAlba3 and PfAlba4.

### Identification of distantly related Alba fold-containing proteins using hidden Markov models

Because PSI-BLAST relies on primary sequence similarity for its searches, it cannot identify divergent protein sequences that adopt the Alba fold. Therefore, we decided to use a Hidden Markov Model (HMM)-based approach to identify all proteins that adopt the Alba fold by implementing Hhpred searches in the MPI bioinformatics toolkit^49^ against the PDB^50^, SCOPe^51,52^, Pfam^53^ and ECOD^54^ databases; the 31 seed sequences listed in Table 1 served as queries. Through this analysis, in addition to known Alba domain-containing proteins, we identified three distant Alba fold-containing proteins: the bacterial sporulation protein VS (SpoVS), the RNA-binding protein YhbY, and the translation initiation factor 3-C (IF3-C). Of these, YhbY and IF3-C have previously been reported to adopt folds similar to the Alba domain^5,55^ whereas there is less evidence for SpoVS, although it is annotated as a member of the Alba superfamily in the InterPro database^4^. In fact, the Alba fold is thought to have evolved within the archaeo-eukaryotic lineage from the YhbY fold^5^.

**Fig. 2:**
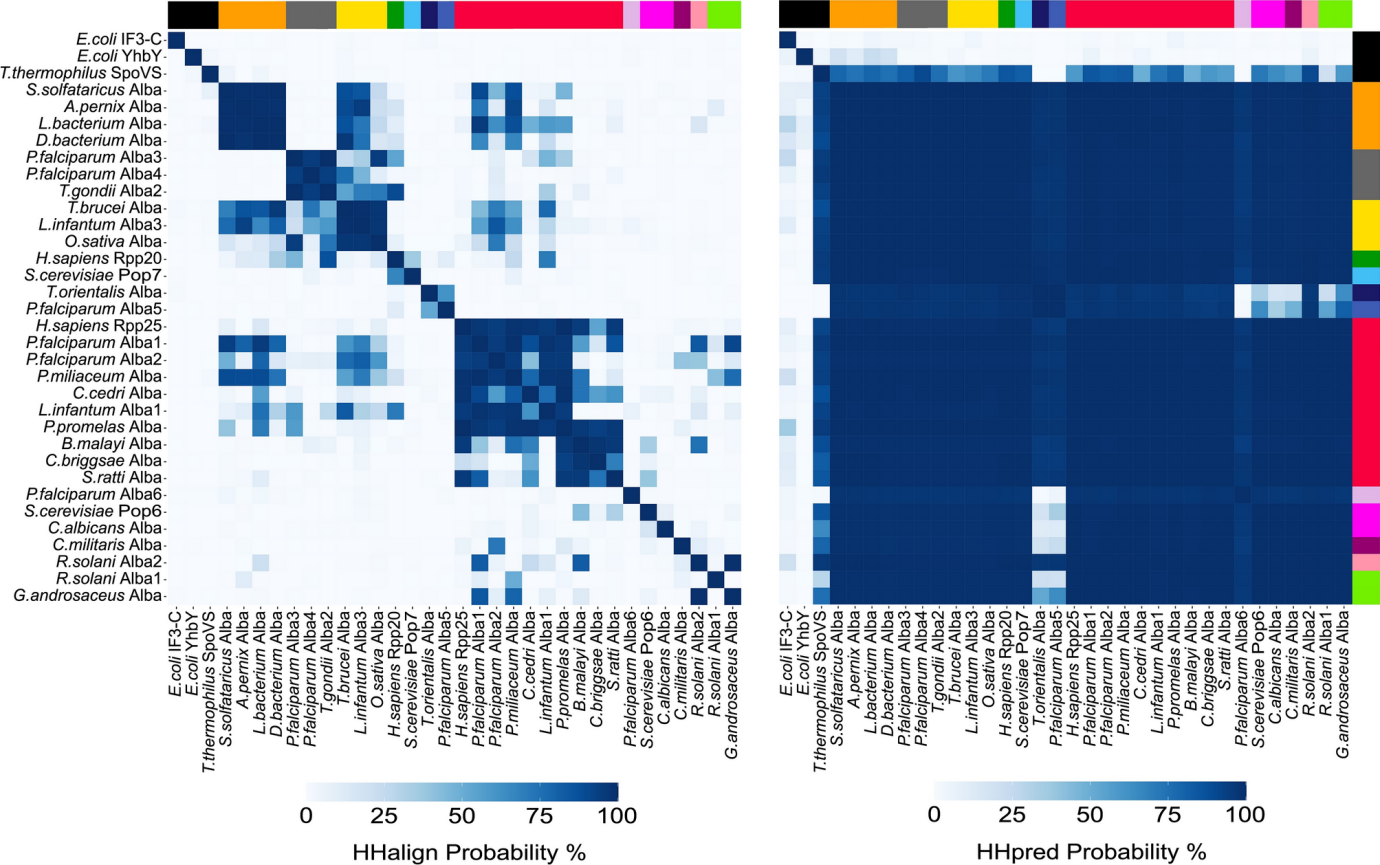
HMM profiles reveal the similarity between Alba domain-containing proteins and SpoVS, a member of the Alba superfamily. Pairwise comparison of primary amino acid sequence (*left panel*) and HMM profiles (*right panel*) of the Alba domain of 31 seed Albas (refer to Table 1 for more details) and three Alba fold-containing proteins, *T. thermophilus* SpoVS (PDB ID:2EK0), *E. coli* YhbY (PDB ID:1LN4) and *E. coli* IF3-C (PDB ID:2IFE). The colour bar represents the SSN cluster to which a given sequence maps to, with black representing the distant homologs identified using Hhpred. Hhalign probability and Hhpred probability values were calculated using the Hhalign module in HH-suite3^56^.

To verify that SpoVS truly adopts the Alba fold, we quantified the pairwise sequence similarity between the 31 seed Alba sequences and YhbY, IF3-C and SpoVS, using the HH-align module in HH-suite3 of the MPI bioinformatics toolkit. We also quantified the pairwise similarity of their profile HMMs based on Hhpred probability percentages. When these values were visualized using a heatmap (Fig. 2; left panel), we observed that the highest level of primary sequence similarity for the 31 seed sequences is at the SSN cluster level, for example, between the archaeal and bacterial Alba proteins, amongst the Alba domain-containing proteins from multicellular eukaryotes, and so on. This was expected since SSN clustering is based on primary sequence identity. In contrast, the distantly related Alba fold-containing proteins, including SpoVS, showed 0.1 to 7.9% sequence similarity to the 31 seed Alba domain proteins and this is probably why they were not identified by PSI-BLAST. Next, upon comparison of the Hhpred probability percentages (Fig. 2; right panel), amongst the Alba fold-containing proteins, SpoVS showed a high Hhpred probability percentage (greater than 90%) with most of the seed Alba sequences, which indicates that it adopts a fold very similar to the Alba domain and can be confidently classified as a member of the Alba superfamily. Contrarily, bacterial YhbY showed a maximum Hhpred probability percentage of 23% with an archaeal Alba, *Aeropyrum pernix* Alba 1, and *Escherichia coli* IF3-C did not show significant HMM profile similarity with any of the proteins, including YhbY and SpoVS.

Focusing on the *P. falciparum* Alba proteins, the Alba domains of PfAlba1 and PfAlba2 shared sequence similarity percentages greater than 50% with Rpp25 and most other Alba domain-containing proteins, be it archaeal, bacterial or eukaryotic, but not with PfAlba3, PfAlba4, PfAlba5, PfAlba6, or select fungal Alba-like proteins that belong to the Fungi Alba2 and Fungi Alba3 clusters (Fig. 2; left panel). PfAlba3 showed a sequence similarity percentage greater than 50% only with PfAlba4, with Alba domains from select unicellular parasitic eukaryotes, and with the Alba-like protein of *Oryza sativa*, while PfAlba4 showed a sequence similarity percentage greater than 50% only with PfAlba3 and with Alba domain-containing proteins of *Trypanosoma brucei brucei*. Interestingly, PfAlba5 shared a sequence similarity percentage greater than 50% only with *Theileria* and *Babesia* Albas while PfAlba6 did not show sequence similarity percentages greater than 50% with any of the proteins included in this analysis. The pattern changed dramatically when we looked at Hhpred probability percentages (Fig. 2; right panel): for PfAlba1, PfAlba2, PfAlba3 and PfAlba4, HMM profile similarity with the remaining Alba seed sequences as well as with SpoVS increased to greater than 90%. For PfAlba5, the probability percentages with most of the seed Albas were high, except for PfAlba6, Pop6, representative sequences from the Fungi Alba1 and Fungi Alba3 clusters, and SpoVS. PfAlba6 showed the most striking shift: although sequence similarities with the other seed Albas were low, HMM profile similarities were high. In fact, the lowest probability percentages were with PfAlba5, *Theileria* Alba and SpoVS. Overall, we infer that although there is low primary sequence conservation amongst Alba domain proteins, they largely adopt the same three-dimensional fold, which can be extrapolated to all of the 15,161 Alba hits identified in this study.

### Structural analysis of Alba domain-containing proteins reveals deviations within the newly identified SSN clusters

**Fig. 3:**
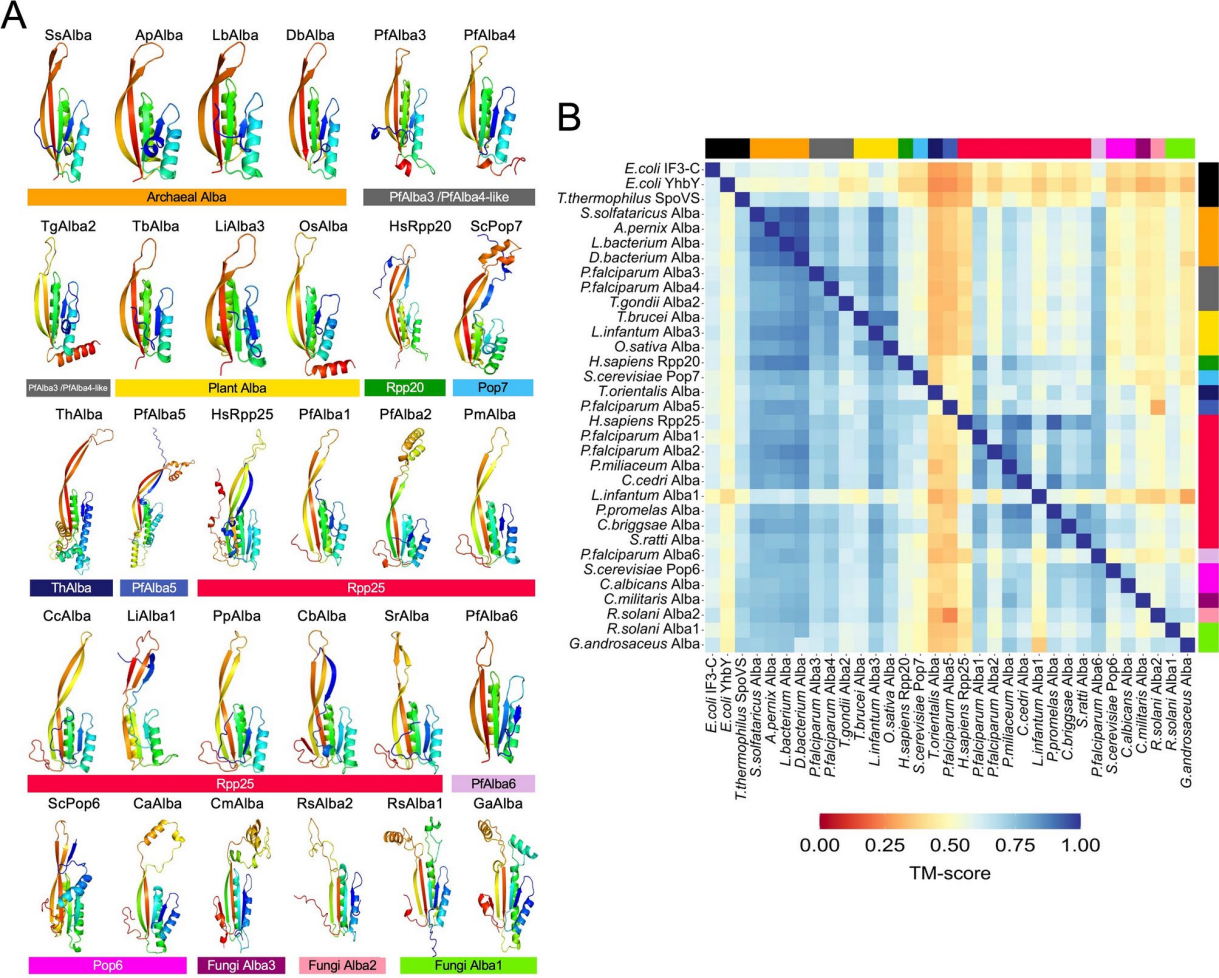
Structural analysis of the Alba-like proteins. (**A**) Structures of the Alba domain of representative seed sequences from each cluster were predicted using RoseTTAFold^57^. The color bar represents the cluster to which each structure belongs. (**B**) Quantitative pairwise comparisons of the 31 representative Alba domain structures and structures of distant homologs were performed using US-align. TM-scores of 0.0–0.30 indicate random structural similarity; TM-scores of 0.5–1.00 indicate that the two proteins adopt generally the same fold.

Further, to explore tertiary structure similarity, we used RoseTTAFold^57^, an ‘accurate’ structure prediction algorithm, to predict the Alba domain structures of the 31 seed Alba sequences and the three distant Alba fold-containing proteins; where available, we also included crystal structures in this analysis. As shown in Fig. 3A, RoseTTAFold was able to predict the Alba domain structures of all seed sequences with high confidence, except for the *Brugya malayi* Alba protein which belongs to the Rpp25 cluster; hence, this structure was not included for further analysis. To assess structural prediction accuracy, the RoseTTAFold-predicted structures of archaeal *Sulfolobus solfotaricus* Alba, *Homo sapiens* Rpp25, *H. sapiens* Rpp20, *S. cerevisiae* Pop6 and *S. cerevisiae* Pop7 were compared to their respective crystal structures using US-align^58^. The resulting TM-align scores were greater than 0.9 indicating high levels of tertiary structure identity (Supplementary Fig. S4).

We thereafter inspected the remaining Alba domain structures. At a first glance, all predicted seed Alba structures revealed conservation of the classic Alba fold: a short N-terminal β-sheet followed by an α-helix, a second β-sheet, another α-helix, and two elongated β-sheets at the C-terminus joined by a flexible loop, i.e., β1–α1–β2–α2–β3–β4 (Fig. 3A). Nonetheless, a few structures exhibited deviations from the classic fold (Supplementary Fig. S5) and are highlighted here: (i) When it comes to lower eukaryotic species, Alba domains have elongated C-terminal β-sheets. A specific case is PfAlba5 which is predicted to contain β3 and β4 of length 21–22 amino acids as compared to archaeal Albas which contain 11–15 amino acid-long β3 and β4. (ii) *S. cerevisiae* Pop6 and Pop7 diverge from Rpp25 and Rpp20, respectively, through the presence of an extra α-helix and β-sheet at the N-terminus*.* (iii) The Alba domains of PfAlba3, PfAlba4 and plant Albas contain an extra C-terminal α-helix which is absent in Rpp20. (iv) The newly identified fungal Alba proteins (Fig. 1B; fluorescent green, peach and magenta clusters) appear to have extra β-sheets and α-helices between β3 and β4 which loop out of the Alba fold.

Subsequently, in a pairwise manner, we compared the RoseTTAFold-predicted structures of the seed Albas to each other or to the crystal structures of SpoVS, YhbY and IF3-C using US-align (Fig. 3B). The TM-align scores of all Alba domain structures relative to the archaeal Alba domain were greater than 0.7, indicating strong tertiary structure identity (Fig. 3B). However, the pairwise TM-align scores for some of the other comparisons were lesser than 0.7, for instance when PfAlba5 and *Theileria* Alba were used as the reference sequence for comparison. Notably, for all seed Albas, the TM-align scores relative to SpoVS, YhbY and IF3-C ranged from 0.4 to 0.6, indicating the common topology that these proteins adopt, despite having very little sequence similarity. This supports the evolutionary relatedness of SpoVS, YhbY and IF3-C to the Alba domain. Lastly, the examination of the electrostatic potential surface map of the predicted Alba seed structures revealed the presence of a highly electropositive pocket, across the board, that most likely corresponds to the site of nucleic acid binding (Supplementary Fig. S6).

### The Alba dimer interface shows the highest amino acid sequence conservation

**Fig. 4:**
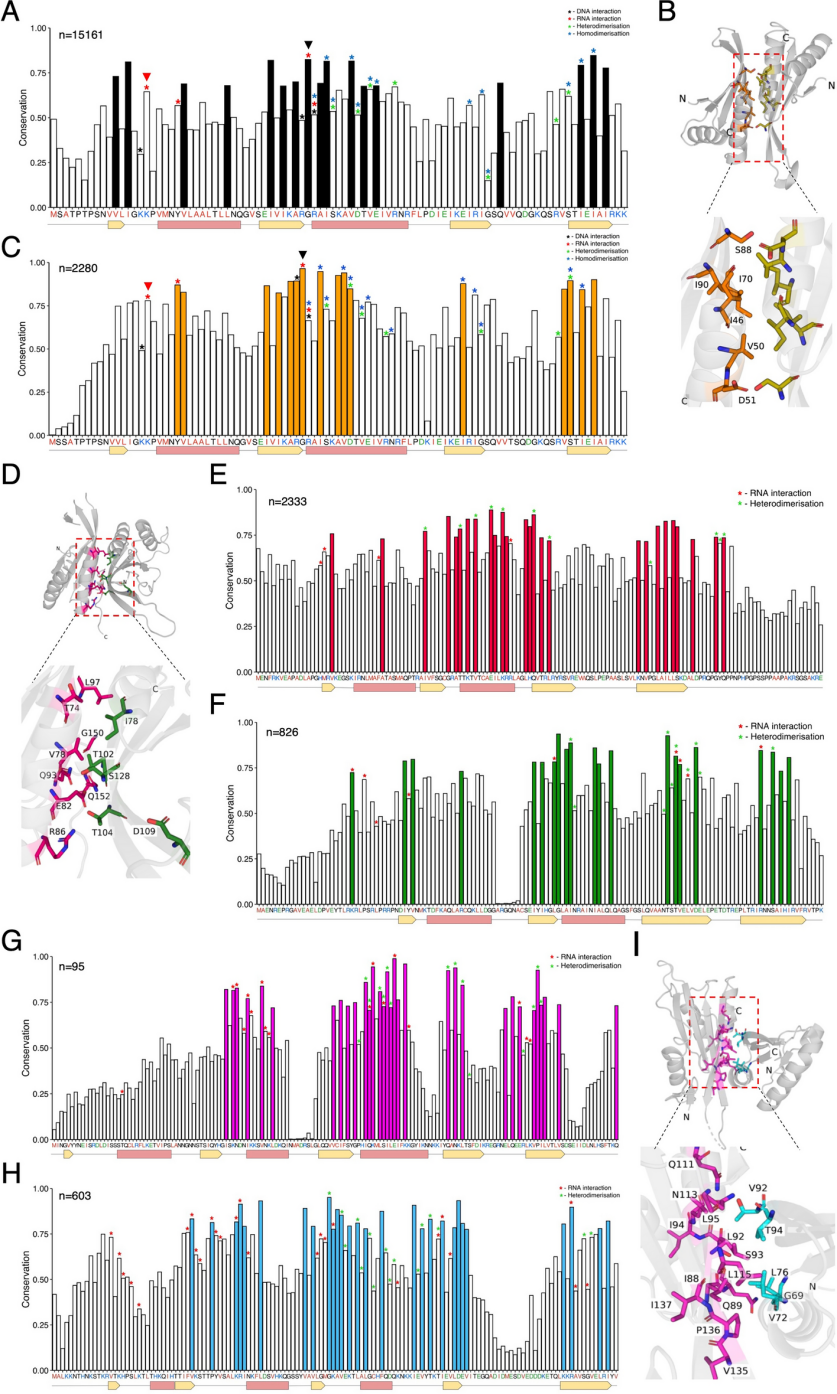
Residues at the dimer interface of Alba homo-/hetero-dimers are significantly conserved. (**A**) Amino acid sequence conservation of all 15,161 Alba homologs was measured relative to an archaeal Alba sequence (Alba1 of *S. solfotaricus*), represented along the X-axis. (**B**) Crystal structure of the dimer interface of the Alba1:Alba2 heterodimer from *S. solfotaricus* (PDB: 2BKY^12^) is shown, with key residues highlighted. (**C**) Conservation of 2280 sequences present in the archaeal Alba cluster was measured relative to Alba1 of *S. solfotaricus*, represented along the X-axis. (**D**) Crystal structure of the dimer interface of human Rpp20-Rpp25 in complex with the P3 stem loop of RNase MRP RNA (PDB: 6LT7^26^) is shown. (**E,F**) Amino acid conservation of protein sequences present in the **(E)** Rpp25 (n = 2333) and **(F)** Rpp20 (n = 826) clusters, respectively. The human homologs are represented along the X-axis. (**G,H**) Amino acid conservation of the proteins present in the **(G)** Pop6 (n = 95) and **(H)** Pop7 (n = 603) clusters, respectively. The *S. cerevisiae* homologs are represented along the X-axis. (**I**) Crystal structure of the dimer interface of *S. cerevisiae* Pop6/Pop7 in complex with the P3 region of the RNase MRP RNA (PDB: 3IAB^60^) is shown. For parts (**A,C,E,F,G,H**) conserved residues of at least one standard deviation more than the mean conservation across the alignment are shown as colour-filled bars. *Highlights functionality of select residues as per the key provided in the figure. In the consensus sequence (X-axis), red represents hydrophobic residues, blue represents positively charged residues, and green represents negatively charged residues. The protein secondary structure is plotted along the X-axis with α-helices coloured in pink and β-sheets in yellow as per the corresponding crystal structure.

We next looked for sequence conservation amongst all 15,161 Alba domain-containing proteins identified in this study relative to archaeal Alba using Scorecons^59^. Additionally, to infer the functional relevance of conservation, we mapped these amino acid residues onto the crystal structure of archaeal Alba(s) in the presence or absence of DNA and/or RNA (PDB IDs:2BKY, 6LT7 and 3IAB^12,26,60^). As seen in Fig. 4A-B, for the 15,161 Alba domain proteins, β2, α2 and β4 contain a higher number of conserved residues, a majority of which are important for dimerization and to a lesser extent, nucleic acid interaction. This pattern of conservation is also observed within the archaeal Alba cluster (Fig. 4C). Of note, the Gly43 residue of archaeal Alba, known for its involvement in RNA binding^9^, showed significant conservation in all Albas. In contrast, the Lys16 residue, subject to acetylation in archaeal Alba and a purported regulator of DNA binding activity^2^, exhibited poor conservation (Fig. 4A-C).

Given the availability of RNA-bound crystal structures of the Rpp20-Rpp25 and Pop6/Pop7 heterodimers, we further looked at sequence conservation within these sub-groups and mapped conserved residues onto the crystal structure. In the Rpp20-Rpp25 heterodimer (PDB: 6LT7^26^), we observed a similar pattern as above, wherein the residues responsible for heterodimerization exhibited a high degree of conservation along with the equivalent of archaeal Alba Gly43 (Fig. 4D–F). Conversely, in the case of the Pop6/Pop7 complex (PDB: 3IAB^60^), residues involved in both RNA interaction and heterodimerization display significant conservation (Fig. 4G–I).

Lastly, sequence conservation analysis for Alba clusters lacking crystal structures (Supplementary Fig. S7) revealed that in the PfAlba3/PfAlba4 cluster, only β2, including the glycine corresponding to archaeal Gly43, exhibits high conservation, while within the plant Alba cluster, β2 and α2 show high conservation, but not β4. Remarkably, conservation scoring of the Fungi Alba2 cluster resulted in the detection of a putative Tubby-like domain (TULP) in conjunction with the Alba domain in all 380 members (Supplementary Fig. S7). The TULP domain is postulated to act as a transcription factor^61^, and may diversify the functionality of its associated Alba domain. Taken together, our analysis suggests that the dimer interface—for either homo- or hetero-dimerization—is the most highly conserved for experimentally characterized Albas. This can be generalised to understand the functionality of the newly identified Alba domain-containing proteins and the impact of homo- or hetero-dimerization on protein function.

### Phylogenetic analysis of Alba-like proteins

**Fig. 5:**
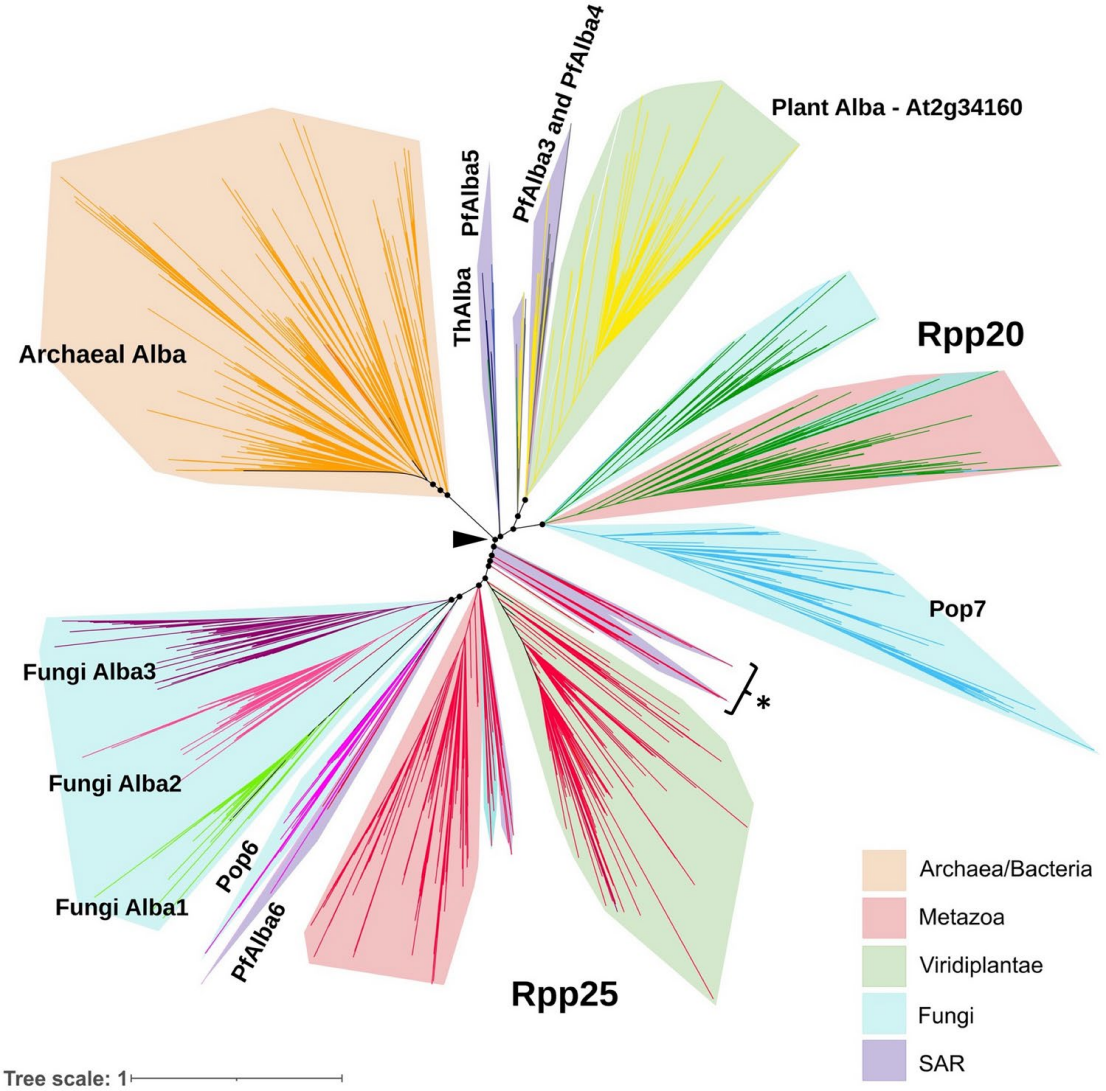
Phylogenetic reconstruction of Alba relatedness. A maximum likelihood tree was built using a representative set of 4666 Alba homologs, which were obtained by clustering the 15,161 hits at a pairwise sequence identity threshold of 70%. Only branch points with an aBayes branch support value greater than 0.75 are represented, resulting in the retention of 4585 Alba homologs in the final tree. The branches are colored based on the cluster information from SSN analysis. Coloured ranges depict different kingdoms. The arrowhead denotes the branchpoint for divergence of Rpp20- and Rpp25-like proteins. *Highlights the branch containing PfAlba1 and PfAlba2. *SAR* Supergroup of Protists containing Stramenopiles, Alveolata and Rhizaria.

Thus far, SSN analysis has provided a comprehensive understanding of the inter-relatedness amongst Alba homologs at the primary sequence level, while secondary and tertiary structure analyses have revealed structure–function relationships for the 13 Alba sub-groups. To obtain insights into evolutionary relatedness, we constructed a maximum-likelihood phylogenetic tree using a selected set of 4666 representative sequences from the 13 SSN-derived Alba clusters, including all 31 seeds. Because Alba sequences are extremely divergent, with pairwise sequence identity as little as 10%, we were unable to find a suitable outgroup and hence, we built an unrooted tree (Fig. 5). The branching of the tree independently supported the clustering observed in SSN analysis, with each major Alba subgroup demonstrating a significant branching probability. Nonetheless, a few deviations were evident, which are discussed below.

Considering the archaeal Albas to be the most ancient members of this protein family, we observed a single branchpoint from which the Rpp20- and Rpp25-like clades diverged (Fig. 5; marked with an arrowhead). Within the former, the earliest diverging branch was comprised of PfAlba5-like proteins and the ThAlbas. Given that these proteins formed separate clusters in the SSN (Fig. 1B; blue and dark blue), we were surprised to find that they were not distinguishable phylogenetically. The second diverging branch in the Rpp20-like clade was comprised of PfAlba3/PfAlba4-like proteins and plant Albas, while the third branch included Rpp20 proteins from fungi, metazoa as well as fungal homologs of Pop7, as separate groups. To a large extent, these branches reflected the clustering observed in the SSN; the only exception was the divergence of fungal and metazoan Rpp20 homologs. On the other hand, within the Rpp25-like clade, the earliest diverging branch was comprised of PfAlba1/PfAlba2 homologs from the Stramenopiles-Alveolata-Rhizaria or SAR taxonomic group followed by distinct branches containing plant and metazoan Rpp25-like proteins (Fig. 5), all of which constituted the red cluster in the SSN of Fig. 1B. This was unexpected and we attributed it to the identity threshold of 55% that we had chosen for SSN clustering. Indeed, when the SSN threshold was increased to 65% identity, we observed that PfAlba1/PfAlba2 and the plant Rpp25-like proteins formed independent clusters which shared edges with a metazoan Rpp25 cluster (data not shown). Other notable features of the phylogenetic tree include the diversification of Pop6-like and PfAlba6-like proteins from a common ancestor within the Rpp25-like clade, branching of the newly identified fungal Albas from a single node in the Rpp25-like clade, and the lack of a single branch for bacterial Albas within the archaeal clade. This latter observation supports the acquisition of Albas by bacteria through horizontal gene transfer. Overall, phylogenetic analysis provided a timeline for the genetic divergence of the Alba domain within eukaryotes.

To gain insights into the potential time periods when the Alba domain may have diversified, we lastly generated a time divergence tree (Supplementary Fig. S8). Spanning the evolutionary history of the Alba domain over the past 2000 million years, the timetree revealed the ancient nature of PfAlba3/PfAlba4 and plant Albas, which diverged from a common ancestral protein around 1800 million years ago (MYA). This was followed by the divergence of Rpp20-like and Rpp25-like proteins approximately 1600 MYA with the most recent divergence being the newly clustered fungal Albas approximately 500–1000 MYA. Overall, the timetree analysis reflected a birth-and-death model of Alba gene evolution, where multiple lineage-specific Alba duplications occurred, particularly in protozoan parasites like *P. falciparum* and *Leishmania*, while complex organisms like *H. sapiens* retained only a few of these genes.

## Discussion

For a given protein domain, amino acid sequences change over time to adapt to physiological and environmental stresses. These changes could either be single amino acid substitutions, additions and deletions, or the incorporation of small peptide units into the basic protein scaffold, all of which increase functionality and provide a fitness benefit^62–64^. Nonetheless, despite the accumulation of changes, it has been observed that a given domain largely retains its three-dimensional functional fold, even in evolutionarily divergent organisms. To understand this phenomenon in detail, researchers have begun performing in depth studies of sequence, structural and functional relationships of ubiquitous proteins, domains and/or folds such as the histone fold, SH3 and OB domains in ribosomal proteins, P-loop NTPases and Rossmann-fold enzymes, glycosyltransferases, ESCRT systems, etc.^36,65–69^, in turn tracing their evolutionary origin. But, analyses of more specialized protein domains lag behind. Here, we use a combination of exhaustive database searches, SSN creation, structural comparison, and phylogenetic reconstruction to understand the inter-relatedness and evolutionary dynamics of the DNA/RNA-binding Alba domain, highlighting its versatility and importance to essential biological processes across all kingdoms of life.

Firstly, by building an SSN containing more than 15,000 Alba domain-containing proteins from different taxonomic lineages, we identified 13 distinct Alba clusters (Fig. 1B). Subsequent phylogenetic analysis (Fig. 5) provided an evolutionary framework for these groupings: for instance, in the SSN, the archaeal Albas clustered with a very small set of Alba domain proteins from bacteria, amongst which 13% mapped to the phylum Proteobacteria and 27% to the Terrabacteria clade, which includes the ancient phyla Actinobacteria and Firmicutes. Moreover, because a majority of the bacterial hits were derived from metagenome assemblies—*i.e.*, their taxonomic classification contains a level of uncertainty—we postulated that select bacteria may have acquired Alba proteins from archaea through horizontal gene transfer. This was supported by the phylogenetic tree, wherein the bacterial Albas did not fall into a single branch but were spread across different branches within the archaeal clade. On the whole, we can irrefutably conclude that this DNA/RNA-binding domain was not present in the Last Universal Common Ancestor, thus upholding a seminal study which arrived at a similar conclusion by comparing fewer than 70 archaeal Alba, eukaryotic Rpp20-like, and eukaryotic Rpp25-like protein sequences^5^. Of note, the Alba domain of Macronuclear development protein 2 or Mdp2 from the ciliate *Stylonychia lemnae*, which was used by Aravind et al*.* to benchmark Rpp25-like Alba proteins but was not included in our list of Alba seed sequences, falls within the Rpp25-like cluster of the SSN and phylogenetic tree.

Another outcome of SSN and phylogenetic analyses was the identification of lineage-specific diversification of eukaryotic Albas. In fact, nine of the 13 SSN clusters corresponded to protist and fungal taxonomic groups such as the genus *Plasmodium*, order Saccharomycetales or division Basidiomycota, suggesting that these unicellular organisms may have diversified their Alba repertoire to regulate DNA- and RNA-dependent processes required for specific lifestyle adaptations such as parasitism. A case in point is the malaria parasite which contains six Albas mapping to four SSN clusters: Rpp25-like (containing PfAlba1 and PfAlba2), PfAlba3/PfAlba4-like, *Plasmodium* Alba5 and *Plasmodium* Alba6, the latter two clusters being genus-specific (Fig. 1B) and with the most recent diversification, that of PfAlba5, happening 530 MYA in the Cambrian period. Phylogenetic analysis further segregated Rpp25-like PfAlba1 and PfAlba2 into a separate clade, distinct from plant and metazoan Rpp25 proteins, suggesting that PfAlba1 and PfAlba2 functions may not be constituents of Plasmodial RNase P/MRP. This is evidenced by their correlation to processes such as the regulation of translation timing and virulence gene expression in *P. falciparum*^15,16,18,70,71^. Even so, the question remains, what is the nature of the RNase P/MRP complex in *Plasmodium* sp.? In humans and budding yeast, it is well-established that Rpp25/Pop6 and Rpp20/Pop7 carry out similar functions in the context of the RNase P/MRP complex during 5ʹ tRNA processing^72–77^ despite their sequence divergence. In contrast, in *A. thaliana* and *T. brucei*, the ribonucleoprotein RNase P complex is substituted by a proteinaceous RNase P, PRORP, for tRNA processing^78^. Whether *Plasmodium* employs the latter strategy or whether PfAlba5 and PfAlba6 take on the roles of Rpp20/Pop7 and Rpp25/Pop6, respectively, remains to be seen; this could be addressed by systematically analyzing the RNA and protein interactomes of each PfAlba, using immunoprecipitation followed by high throughput sequencing and mass spectrometry, respectively.

A second case in point is *T. gondii*, a parasite closely related to *Plasmodium.* Its four Alba-domain containing proteins segregate into four distinct SSN groups: Rpp25-like (TgAlba1), Pop7 (ToxoDB ID/NCBI ID: TGME49_245520/XP_018634887), Rpp20-like (ToxoDB ID/NCBI ID: TGME49_217665/XP_018634862), and PfAlba3/PfAlba4-like (TgAlba2). Experimental characterization of TgAlba1 and TgAlba2 showed that both proteins co-localize in vivo and are involved in translational control of gene expression^24^. Whether they function as a heterodimer is not known, neither is the function of the remaining TgAlbas, which could be an avenue of future research, particularly in the context of host infection. A last, but contrasting case in point, is with regards to plant Albas. Crop species such as *O. sativa, Zea mays, Sorghum bicolor*, *Cicer arietinum,* and *Vitis vinifera*, and the model plant *A. thaliana* express anywhere from six to 20 different Alba domain-containing proteins^79^. However, our analysis placed these proteins into just two clades, one that is Rpp20-like and the other that is Rpp25-like (Fig. 5). This suggests that, to adapt to the ever-changing environmental conditions of the Proterozoic eon, unicellular eukaryotes may have tolerated significant amino acid substitutions in the Alba domain without compromising on the structural integrity of the Alba fold.

Subsequent sequence conservation analysis across the 13 SSN clusters did not identify a single invariant amino acid residue that participated in nucleic acid recognition. Instead, heterodimerization emerged as a universal feature of the Alba protein family, substantiated by the high conservation of the dimer interface (Fig. 4, Supplementary Fig. S7). The importance of this phenomenon for Alba function is already well-established: for instance, in archaea, the Alba1:Alba2 heterodimer showed a greater level of DNA compaction as compared to the homodimer of either Alba^12,80^. Thus, by differentially regulating the expression of Alba1 and Alba2, chromatin compaction in archaea could be modulated, in turn altering gene expression. In humans, the Rpp20–Rpp25 heterodimer functions as a single unit, with heterodimerization being a prerequisite for interaction with the P3 RNA of RNAse P^27^. In yeast, a similar prerequisite for Pop6–Pop7 heterodimer formation has been reported^60,81^. In *A. thaliana*, AtAlba1 and AtAlba2, which cluster with the plant Alba-like family in the SSN, bind to genic R-loops as heterodimers and maintain genome stability^32^. Taken together, these studies suggest that Alba proteins recognise bipartite motifs in nucleic acids. Lastly, in addition to heterodimerization, homodimerization has also been demonstrated for select Albas* in vitro*^7,15,20^ though its functional significance remains to be seen. Moreover, in organisms that express more than two Alba proteins, the permutations of heterodimers that can form may increase dramatically, which could impart divergent functions to this protein family. A future avenue of research could involve mutating the conserved amino residues of the dimer interface identified here and studying its effect on dimerization using biochemical assays as well as *in cellulo* in model organisms such as *S. cerevisiae* and *A. thaliana*.

At the global level, all of the 15,161 proteins that we obtained through our exhaustive searches adopted the β1–α1–β2–α2–β3–β4 Alba fold, although subtle variations were evident. Eukaryotes especially contain longer Alba domains with additional α-helices or extended β-sheets, which may allow for tight regulation of their activity and/or enhance their RNA targeting capacity. This is particularly evident in PfAlba5, which contains longer β3 and β4 sheets compared to archaeal Albas (Supplementary Fig. S5). Because amino acid residues in β3 and β4 are known to directly contact RNase P/MRP RNA, changes in the length of these β-sheets may allow PfAlba5 to recognize non-canonical RNA substrates. Further, the structural variations between PfAlba3/PfAlba4 and human Rpp20 (Fig. 3A) confirm that these Albas are not homologs of Rpp20 and may have unique functions in *P. falciparum*. For example, a recent study demonstrated that PfAlba3 has endonuclease activity in vitro^19^. Taken together with the observation that archaeal Albas also possess nickase activity^82^, the early divergence of PfAlba3/PfAlba4 (Supplementary Fig. S8) may have allowed them to retain select structural features that enable phosphodiester bond cleavage, which remain to be explored. Lastly, a group of structurally deviant Alba domain proteins belong to the fluorescent green, peach and magenta fungal clusters in Fig. 1B. The extra loops and α-helices found between β3 and β4 of the Alba fold may either support the recognition of diverse RNA structures or may help these proteins to form heterogeneous ribonucleoprotein complexes. However, because these are predicted structures, we cannot conclusively comment on the functional implications of these structural variations. Nevertheless, complementation assays in the budding yeast *S. cerevisiae* could serve as a starting point to determine whether these new fungal Albas rescue the function of yeast Pop6 or Pop7; if they do, the importance of the additional loops and α-helices could be explored by mutational analysis.

The overall lack of any invariant residues across the Alba domain classes identified here indicates the extensive primary sequence innovations that this domain has undergone. A protein fold is said to be ‘innnovable’ when the structural scaffold, which provides robustness and stability to the fold, and the active site residues, which recognise substrates, are highly separated^83^; this enables substrate diversification, which can consequently impart diverse functions. In the case of the Alba domain, the scaffold remains highly conserved, thus retaining a highly electropositive surface for nucleic acid binding (Supplementary Fig. S7). However, by varying the sequence as well as length of loops between the structural elements over time, the domain may have gained the ability to recognise different types of nucleic acids and sequence motifs, in addition to other biomolecules with electronegative surfaces such as RNA-binding proteins with intrinsically disordered regions. Indeed, the innovability of this domain can be observed in organisms such as *Plasmodium* where there is lineage-specific evolution, and also in the most recently diverged fungal Albas, which have a characteristic extension of α2 and new loops in between β3 and β4. Additional functions may be imparted by the co-occurring domains like TAXi_N-TAXi_C, HHH_5, TULP, and RGG motifs. These domains are implicated in biological processes related to transcriptional regulation, RNA binding, protein folding and response to stress. In fact, the RGG motif, frequently found in RNA-binding proteins involved in stress granule formation^84^, further supports a role for Alba domain-containing proteins in maintaining RNA homeostasis under stress conditions.

Collectively, our findings strongly indicate the evolutionary trajectory of Alba proteins, transitioning from sequence-independent NAPs in archaea, primarily responsible for genomic organization, to specialized proteins that exhibit selective recognition of specific nucleic acids in a particular protein complex or pathway over a span of 1000 million years. Notably, nucleic acid binding activity has been retained across all members of the Alba superfamily, underscoring its pivotal role in diverse biological contexts. This can be correlated to other protein families such as bacterial integration host factor or IHF, a homolog of histone HU which evolved to become a sequence-specific transcriptional activator at certain genetic loci while maintaining genome organization capability at others^85^. Another example is the chromatin architectural protein TK0471 from the archaea *Thermococci kodakarensis,* which was identified to be homologous to the TrmB transcription factor, a specialised regulator of the *mal* operon^86^. Lastly, two archaeal proteins, Smj12 and Abfr1, which non-specifically bind to and condense DNA^87,88^, are both members of the Lrs14 family of specialised transcription factors^89^.

Our sequence-structure analysis serves as a starting point for inferring the functionality of a range of Alba proteins. If we consider that proteins which perform the same function most likely share similar sequences, and hence cluster together, then we can assign specific functions to select Alba clusters. Additionally, we can predict the heterodimer pairs that could form in organisms with three or more Alba domain proteins. For example, the primary functions of proteins in the Rpp25-like, Rpp20-like, Pop6 and Pop7 clusters is most likely RNase P/MRP activity, with additional moonlighting functions such as the regulation of telomerase activity; the resulting heterodimer pairs could be arrived at in a similar fashion. On the other hand, the major functions of proteins in the plant Alba-like cluster, which contains entries from plants as well as Discoba, could be translational regulation and maintenance of genome stability. Extrapolating further, all proteins in the PfAlba3/PfAlba4-like cluster may possess site-driven endonuclease activity in the presence of divalent metal ions in vitro, as was recently demonstrated for PfAlba3^19^, although the in vivo significance of nuclease activity remains to be explored. Nonetheless, we cannot rule out that the function of a given Alba domain-containing protein may be determined not just by the Alba domain, but by its co-occurring domains, post-translational modifications, and/or sub-cellular localisation. Further studies of individual Albas from diverse clusters and clades will be required to dissect out the range of cellular processes controlled by this protein family.

In conclusion, our investigation into the evolutionary dynamics and functional diversity of the Alba protein domain sheds light on its remarkable adaptability and significance across the biological spectrum, particularly in the context of genome maintenance and RNA biology. Moreover, our study provides a foundational framework for predicting functional roles and potential heterodimerization partners within the Alba protein family, paving the way for future investigations into the cellular processes controlled by this protein domain. To the best of our knowledge, this study is the first of its kind for a specialised DNA/RNA/DNA:RNA-binding domain, and paves the way for similar studies into innovable protein domains.

## Materials and methods

### Gathering Alba-domain containing proteins

A list of Alba domain-containing proteins from various domains of life was compiled from literature searches, and the sequences of their Alba domains extracted in FASTA format; the average length of the domain was found to be 90 to 120 amino acids in archaea, and 100 to 160 amino acids in eukaryotes. Homology modeling was used to confirm that the extracted sequences corresponded solely to the Alba fold, which were then used as ‘seed’ sequences for preliminary searches in PSI-BLAST^34^ against the NCBI non-redundant (nr) protein database^35^ with an E-value cut-off of 10 and a total of five iterations (PSI-BLAST parameters: -num_iterations = 5 -max_target_seqs = 10,000 -evalue = 10 -num_threads 8 -outfmt ‘6 qseqid sseqid stitle qstart qend sstart send pident evalue qcovs gaps bitscore length sseq sacc sgi staxid ssciname scomname sskingdom’). The resulting list of ‘hits’ was filtered at 90% maximum pairwise identity using MMseqs2^38^ and clustered using CLANS^37^ with default parameters (MMseqs2 Web Server Parameters—Minimum_sequence_identity = 90 -Minimum_Alignment_Converage = 80 -Clustering_Mode Normal- > slow_sensitive). Clusters were defined based on a p-value cutoff of 1e-4. For clusters that did not contain any of the initial seed sequences, a consensus sequence was created and the ‘hit’ with highest sequence identity to the consensus was chosen as the best representative. These representative sequences were manually investigated to confirm the presence of the Alba domain using HHpred searches^90^ and RoseTTAFold structure predictions^57^. Clusters that passed these investigations were retained and their representative sequences used for a second round of PSI-BLAST; all other singletons in the CLANS cluster map were removed. This process was iterated until no new clusters were discovered. The resulting final list of seed sequences was used to query the September 2021 release of the NCBI nr protein database by performing PSI-BLAST with an E-value cut-off of 10 and a total of five iterations. The hits were clustered in CLANS after filtering for a maximum pairwise identity of 90% using MMSeqs2 and clusters defined based on a p-value cutoff of 1e−4. The Krona visualisation tool^91^ was used to examine the extent of distribution of Alba domain-containing proteins across all taxonomic groups and in each cluster.

### Network building and visualisation

The Enzyme Function Initiative’s (EFI’s) web resources^45^ were used to generate the Alba Sequence Similarity Network (SSN), which was built by performing an all-against-all BLAST to calculate the pairwise similarities of all input sequences. A FASTA file containing 8390 sequences was used as input, and an E-value of 4 was used to calculate the SSN edge alignment score similarities, as described in the EFI web resources (https://efi.igb.illinois.edu/efi-est/). Because visualising the entire network was computationally expensive, a representative network filtered to a maximum pairwise identity of 55 percent using CD-HIT^44^ and containing 5967 nodes and 2,033,164 edges was generated. For network visualisation, the Prefuse force-directed layout in Cytoscape v3.9.1^47^ was used, with edge E-value threshold set to 1E−4. Clusters were defined based on the CLANS map shown in Supplementary Fig. S1.

### Identifying distant homologs using HHpred and pairwise HMM comparison

Using the final list of 31 Alba seed sequences as input, HHpred searches were performed in January 2022 against the PDB mmCIF70, SCOPe70, Pfam-A v35, and ECOD F70 databases, as well as proteomes of some model organisms whose profile HMMs were available in the MPI Bioinformatics toolkit database^49^. This ensured that the searches had converged and that all members of the Alba superfamily as well as distant homologs had been assimilated. Next, for seed sequences and the newly identified distant homologs, pairwise sequence similarity was calculated using the HHalign module in HH-suite3^56^. To create profile HMM for each sequence, three iterations of HHblits^56^ was run against the Uniclust30 database^92^ with an E-value inclusion threshold of 1E−3. The profile HMMs were then pairwise aligned with HHalign to obtain the HHpred probability percentage.

### Protein structure prediction and comparison

RoseTTAFold, an accurate protein structure prediction method based on deep learning^57^, was used to predict the structures of the 31 seed sequences and three distant homologs *Thermus thermophilus* SpoV (PDB ID:2EK0^93^), *Escherichia coli* YbhY (PDB ID:1LN4^94^) and *E. coli* IF3-C (PDB ID:2IFE^95^). Although crystal structures for some of the seed sequences were available, all structures were predicted using RoseTTAFold to ensure uniformity. TM-scores were calculated using US-align^58^ for pairwise comparisons of all predicted structures, and the accuracy of the predicted structures was also compared to crystal structures wherever available. All models were visualised in PyMol 2.5.2 (Schrödinger, LLC, 2015)^96^, retrieved from http://www.pymol.org/pymol. For all predicted structures, the electrostatic potential surface charges were calculated using the APBS Electrostatic Potential plugin^97^ in PyMol.

### Sequence conservation and phylogenetic analysis

All sequence alignments were performed using the MAFFT v.7.490 program^98^ with the L-INS-i algorithm. Conservation of amino acid residues was quantified using the Scorecons server^59^ against the sequences whose crystal structures were available^8,25,60,99,100^. For the phylogenetic reconstruction, sequences from each cluster in the CLANS cluster map, except for clusters with fewer than 30 sequences, were extracted and filtered using MMseqs2 at a maximum pairwise identity of 70% to obtain a list of 4666 sequences. The alignment was then trimmed using Goalign ‘clean sites’ option^101^. A maximum likelihood tree was inferred using IQTree v2.1.2^102^ with 3000 bootstrap iterations and automatic substitution model selection^103^. For in-depth inferences, -nstop was set to 300 and -pers to 0.3. iTOL^104^ was used to visualise and annotate the tree. For generating the Timetree, seed sequences excluding bacterial Albas were aligned using MAFFT v.7.490^98^ with the L-INS-I algorithm followed by maximum-likelihood tree estimation using IQTree v2.1.2^102^ with 1000 bootstrap iterations and automatic substitution model selection^103^. Rel-Time algorithm^105,106^ implemented in MEGA 11^107^ was used to infer the divergence rate based on the time calibration constraints for the three branches obtained from the TimeTree 5 Database^108^.

## Supplementary Information

Supplementary Information.

## Data Availability

Proteins identified as Alba domain-containing from NCBI PSI-BLAST have been collated into a R Shiny app which is available at: http://plasmobees.shinyapps.io/Alba_domain_containing_proteins. Taxonomic information of each cluster is available as an interactive Krona chart at: https://github.com/jaiganeshjg/Alba. All scripts, commands and result files for each figure have been deposited in Github (https://github.com/jaiganeshjg/Alba_manuscript).

## References

[1] Visweswariah, S. S. & Busby, S. J. W. Evolution of bacterial transcription factors: How proteins take on new tasks, but do not always stop doing the old ones. *Trends Microbiol.* **23**(8), 463–467. 10.1016/j.tim.2015.04.009 (2015). 26003748 10.1016/j.tim.2015.04.009

[2] Bell, S. D., Botting, C. H., Wardleworth, B. N., Jackson, S. P. & White, M. F. The interaction of Alba, a conserved archaeal chromatin protein, with Sir2 and its regulation by acetylation. *Science* **296**(5565), 148–151. 10.1126/science.1070506 (2002). 11935028 10.1126/science.1070506

[3] Lurz, R., Grote, M., Dijk, J., Reinhardt, R. & Dobrinski, B. Electron microscopic study of DNA complexes with proteins from the Archaebacterium *Sulfolobus acidocaldarius*. *EMBO J.* **5**(13), 3715–3721 (1986). 16453745 10.1002/j.1460-2075.1986.tb04705.x PMC1167416

[4] Paysan-Lafosse, T. et al. InterPro in 2022. *Nucleic Acids Res.* **51**(D1), D418–D427. 10.1093/nar/gkac993 (2023). 36350672 10.1093/nar/gkac993 PMC9825450

[5] Aravind, L., Iyer, L. M. & Anantharaman, V. The two faces of Alba: The evolutionary connection between proteins participating in chromatin structure and RNA metabolism. *Genome Biol.* **4**(10), 1–9. 10.1186/gb-2003-4-10-r64 (2003). 10.1186/gb-2003-4-10-r64 PMC328453 14519199

[6] Dijk, J. & Reinhardt, R. The structure of DNA-binding proteins from Eu- and Archaebacteria. In *Bacterial Chromatin* (eds Gualerzi, C. O. & Pon, C. L.) 185–218 (Springer, 1986).

[7] Wardleworth, B. et al. Structure of Alba: An archaeal chromatin protein modulated by acetylation. *EMBO J.* **21**(17), 4654–4662. 10.1093/EMBOJ/CDF465 (2002). 12198167 10.1093/emboj/cdf465 PMC125410

[8] Liu, Y. et al. The Sac10b homolog in *Methanococcus maripaludis* binds DNA at specific sites. *J. Bacteriol.* **191**(7), 2315–2329. 10.1128/JB.01534-08 (2009). 19168623 10.1128/JB.01534-08 PMC2655493

[9] Guo, L. et al. Biochemical and structural insights into RNA binding by Ssh10b, a member of the highly conserved Sac10b protein family in Archaea. *J. Biol. Chem.* **289**(3), 1478–1490. 10.1074/jbc.M113.521351 (2014). 24307170 10.1074/jbc.M113.521351 PMC3894330

[10] Guo, R., Xue, H. & Huang, L. Ssh10b, a conserved thermophilic archaeal protein, binds RNA in vivo. *Mol. Microbiol.* **50**(5), 1605–1615. 10.1046/j.1365-2958.2003.03793.x (2003). 14651642 10.1046/j.1365-2958.2003.03793.x

[11] Zhang, N., Guo, L. & Huang, L. The Sac10b homolog from *Sulfolobus islandicus* is an RNA chaperone. *Nucleic Acids Res.* **48**(16), 9273–9284. 10.1093/nar/gkaa656 (2020). 32761152 10.1093/nar/gkaa656 PMC7498313

[12] Jelinska, C. et al. Obligate heterodimerization of the archaeal Alba2 protein with Alba1 provides a mechanism for control of DNA Packaging. *Structure* **13**(7), 963–971. 10.1016/j.str.2005.04.016 (2005). 16004869 10.1016/j.str.2005.04.016

[13] Jelinska, C., Petrovic-Stojanovska, B., Ingledew, W. J. & White, M. F. Dimer-dimer stacking interactions are important for nucleic acid binding by the archaeal chromatin protein Alba. *Biochem. J.* **427**(1), 49–55. 10.1042/BJ20091841 (2010). 20082605 10.1042/BJ20091841 PMC2841500

[14] Tanaka, T., Padavattan, S. & Kumarevel, T. Crystal structure of archaeal chromatin protein Alba2-double-stranded DNA complex from *Aeropyrum pernix* K1. *J. Biol. Chem.* **287**(13), 10394–10402. 10.1074/jbc.M112.343210 (2012). 22334696 10.1074/jbc.M112.343210 PMC3322981

[15] Chêne, A. et al. PfAlbas constitute a new eukaryotic DNA/RNA-binding protein family in malaria parasites. *Nucleic Acids Res.* **40**(7), 3066–3077. 10.1093/nar/gkr1215 (2012). 22167473 10.1093/nar/gkr1215 PMC3326326

[16] Goyal, M. et al. Identification and molecular characterization of an Alba-family protein from human malaria parasite *Plasmodium falciparum*. *Nucleic Acids Res.* **40**(3), 1174–1190. 10.1093/nar/gkr821 (2012). 22006844 10.1093/nar/gkr821 PMC3273813

[17] Reddy, B. P. N. et al. A bioinformatic survey of RNA-binding proteins in Plasmodium. *BMC Genom.* **16**, 890. 10.1186/s12864-015-2092-1 (2015). 10.1186/s12864-015-2092-1 PMC4630921 26525978

[18] Vembar, M. et al. The PfAlba1 RNA-binding protein is an important regulator of translational timing in *Plasmodium falciparum* blood stages. *Genome Biol.* **16**, 212. 10.1186/s13059-015-0771-5 (2015). 26415947 10.1186/s13059-015-0771-5 PMC4587749

[19] Banerjee, C. et al. Nuclease activity of *Plasmodium falciparum* Alba family protein PfAlba3. *Cell Rep.* **42**(4), 112292. 10.1016/j.celrep.2023.112292 (2023). 36947546 10.1016/j.celrep.2023.112292

[20] Nag, S. et al. *Plasmodium falciparum* Alba6 exhibits DNase activity and participates in stress response. *IScience* **27**(4), 109467. 10.1016/j.isci.2024.109467 (2024). 38558939 10.1016/j.isci.2024.109467 PMC10981135

[21] Bevkal, S. et al. An Alba-domain protein required for proteome remodelling during trypanosome differentiation and host transition. *PLoS Pathog.* **17**(1), e1009239. 10.1371/JOURNAL.PPAT.1009239 (2021). 33493187 10.1371/journal.ppat.1009239 PMC7861527

[22] da Costa, K. S. et al. Structural and evolutionary analysis of Leishmania Alba proteins. *Mol. Biochem. Parasitol.* **217**, 23–31. 10.1016/j.molbiopara.2017.08.006 (2017). 28847609 10.1016/j.molbiopara.2017.08.006

[23] Ferreira, T. R. et al. PRMT7 regulates RNA-binding capacity and protein stability in Leishmania parasites. *Nucleic Acids Res.* **48**(10), 5511–5526. 10.1093/nar/gkaa211 (2020). 32365184 10.1093/nar/gkaa211 PMC7261171

[24] Gissot, M. et al. *Toxoplasma gondii* Alba proteins are involved in translational control of gene expression. *J. Mol. Biol.* **425**(8), 1287–1301. 10.1016/j.jmb.2013.01.039 (2013). 23454356 10.1016/j.jmb.2013.01.039

[25] Chan, C., Kiesel, B. & Mondragón, A. Crystal structure of human Rpp20/Rpp25 reveals quaternary level adaptation of the Alba scaffold as structural basis for single-stranded RNA binding. *J. Mol. Biol.* **430**(10), 1403–1416. 10.1016/J.JMB.2018.03.029 (2018). 29625199 10.1016/j.jmb.2018.03.029 PMC5951771

[26] Yin, C., Bai, G., Zhang, Y. & Huang, J. Crystal structure of human RPP20-RPP25 proteins in complex with the P3 domain of lncRNA RMRP. *J. Struct. Biol.* **213**(2), 107704. 10.1016/j.jsb.2021.107704 (2021). 33571640 10.1016/j.jsb.2021.107704

[27] Hands-Taylor, K. L. D. et al. Heterodimerization of the human RNase P/MRP subunits Rpp20 and Rpp25 is a prerequisite for interaction with the P3 arm of RNase MRP RNA. *Nucleic Acids Res.* **38**(12), 4052–4066. 10.1093/nar/gkq141 (2010). 20215441 10.1093/nar/gkq141 PMC2896528

[28] Li, Y. & Altman, S. A subunit of human nuclear RNase P has ATPase activity. *Proc. Natl. Acad. Sci. U.S.A.* **98**(2), 441–444 (2001). 11149958 10.1073/pnas.021555498 PMC14605

[29] Jiang, T. & Altman, S. Protein–protein interactions with subunits of human nuclear RNase P. *Proc. Natl. Acad. Sci. U.S.A.* **98**(3), 920–925 (2001). 11158571 10.1073/pnas.021561498 PMC14685

[30] Lemieux, B. et al. Active yeast telomerase shares subunits with ribonucleoproteins RNase P and RNase MRP. *Cell* **165**(5), 1171–1181. 10.1016/j.cell.2016.04.018 (2016). 27156450 10.1016/j.cell.2016.04.018 PMC4874874

[31] Náprstková, A. et al. Characterization of alba family expression and localization in *Arabidopsis thaliana* generative organs. *Int. J. Mol. Sci.* **22**(4), 1–23. 10.3390/ijms22041652 (2021). 10.3390/ijms22041652 PMC7914821 33562109

[32] Yuan, W. et al. ALBA protein complex reads genic R-loops to maintain genome stability in Arabidopsis. *Sci. Adv.* **5**(5), 9040. 10.1126/sciadv.aav9040 (2019). 10.1126/sciadv.aav9040 PMC6520018 31106272

[33] Verma, J. K. et al. OsAlba1, a dehydration-responsive nuclear protein of rice (*Oryza sativa* L. ssp. Indica), participates in stress adaptation. *Phytochemistry* **100**, 16–25. 10.1016/J.PHYTOCHEM.2014.01.015 (2014). 24534105 10.1016/j.phytochem.2014.01.015

[34] Altschul, S. F. et al. Gapped BLAST and PSI-BLAST: A new generation of protein database search programs. *Nucleic Acids Res.* **25**(17), 3389–3402. 10.1093/nar/25.17.3389 (1997). 9254694 10.1093/nar/25.17.3389 PMC146917

[35] Sayers, E. W. et al. Database resources of the national center for biotechnology information. *Nucleic Acids Res.* **50**(D1), D20–D26. 10.1093/nar/gkab1112 (2022). 34850941 10.1093/nar/gkab1112 PMC8728269

[36] Alva, V. & Lupas, A. N. Histones predate the split between bacteria and archaea. *Bioinformatics* **35**(14), 2349–2353. 10.1093/bioinformatics/bty1000 (2019). 30520969 10.1093/bioinformatics/bty1000

[37] Frickey, T. & Lupas, A. N. Phylogenetic analysis of AAA proteins. *J. Struct. Biol.* **146**(1–2), 2–10. 10.1016/j.jsb.2003.11.020 (2004). 15037233 10.1016/j.jsb.2003.11.020

[38] Steinegger, M. et al. *MMseqs2 User Guide* (2021).

[39] Črnigoj, M. et al. Interactions of Archaeal Chromatin Proteins Alba1 and Alba2 with Nucleic Acids PLoS ONE **8**(2) e58237. 10.1371/journal.pone.0058237 (2013) 10.1371/journal.pone.0058237 PMC3585288 23469156

[40] Mani Andreas, J. et al. Alba-Domain Proteins of Trypanosoma brucei Are Cytoplasmic RNA-Binding Proteins That Interact with the Translation Machinery PLoS ONE **6**(7) e22463. 10.1371/journal.pone.0022463 (2011) 10.1371/journal.pone.0022463 PMC3141063 21811616

[41] Dupé Carole, A. & Papadopoulou, D. B. An Alba‐domain protein contributes to the stage‐regulated stability of amastin transcripts in L eishmania Summary Molecular Microbiology **91**(3), 548–561. 10.1111/mmi.2014.91.issue-3 (2014) 10.1111/mmi.12478 24620725

[42] Evelien M. et al. The mRNA-bound proteome of the human malaria parasite Plasmodium falciparum Genome Biology **17**(1) 10.1186/s13059-016-1014-0 (2016) 10.1186/s13059-016-1014-0 PMC4933991 27381095

[43] Guerreiro Elena, A. et al. Genome-wide RIP-Chip analysis of translational repressor-bound mRNAs in the Plasmodium gametocyte Abstract Genome Biology 15(11) 10.1186/s13059-014-0493-0 (2014) 10.1186/s13059-014-0493-0 PMC4234863 25418785

[44] Huang, Y., Niu, B., Gao, Y., Fu, L. & Li, W. CD-HIT suite: A web server for clustering and comparing biological sequences. *Bioinformatics* **26**(5), 680. 10.1093/BIOINFORMATICS/BTQ003 (2010). 20053844 10.1093/bioinformatics/btq003 PMC2828112

[45] Zallot, R., Oberg, N. & Gerlt, J. A. The EFI web resource for genomic enzymology tools: Leveraging protein, genome, and metagenome databases to discover novel enzymes and metabolic pathways. *Biochemistry* **58**(41), 4169–4182. 10.1021/ACS.BIOCHEM.9B00735/SUPPL_FILE/BI9B00735_SI_002.PDF (2019). 31553576 10.1021/acs.biochem.9b00735 PMC7057060

[46] Lipman, D. J., Souvorov, A., Koonin, E. V., Panchenko, A. R. & Tatusova, T. A. The relationship of protein conservation and sequence length. *BMC Evol. Biol.* **2**(1), 1–10. 10.1186/1471-2148-2-20 (2002). 12410938 10.1186/1471-2148-2-20 PMC137605

[47] Shannon, P. et al. Cytoscape: A software environment for integrated models of biomolecular interaction networks. *Genome Res.* **13**(11), 2498–2504. 10.1101/GR.1239303 (2003). 14597658 10.1101/gr.1239303 PMC403769

[48] Goyal, M., Banerjee, C., Nag, S. & Bandyopadhyay, U. The Alba protein family: Structure and function. *Biochim. Biophys. Acta Proteins Proteom.* **1864**(5), 570–583. 10.1016/j.bbapap.2016.02.015 (2016). 10.1016/j.bbapap.2016.02.015 26900088

[49] Gabler, F. et al. Protein sequence analysis using the MPI bioinformatics toolkit. *Curr. Protoc. Bioinform.* **72**(1), 1–30. 10.1002/cpbi.108 (2020). 10.1002/cpbi.108 33315308

[50] Berman, H. M. et al. The protein data bank. *Nucleic Acids Res.* **28**(1), 235–242. 10.1093/nar/28.1.235 (2000). 10592235 10.1093/nar/28.1.235 PMC102472

[51] Chandonia, J.-M. et al. SCOPe: Improvements to the structural classification of proteins—Extended database to facilitate variant interpretation and machine learning. *Nucleic Acids Res.* **50**(D1), D553–D559. 10.1093/nar/gkab1054 (2022). 34850923 10.1093/nar/gkab1054 PMC8728185

[52] Fox, N. K., Brenner, S. E. & Chandonia, J.-M. SCOPe: Structural classification of proteins—Extended, integrating SCOP and ASTRAL data and classification of new structures. *Nucleic Acids Res.* **42**(D1), D304–D309. 10.1093/nar/gkt1240 (2014). 24304899 10.1093/nar/gkt1240 PMC3965108

[53] Mistry, J. et al. Pfam: The protein families database in 2021. *Nucleic Acids Res.* **49**(D1), D412–D419. 10.1093/nar/gkaa913 (2021). 33125078 10.1093/nar/gkaa913 PMC7779014

[54] Cheng, H. et al. ECOD: An evolutionary classification of protein domains. *PLoS Comput. Biol.* **10**(12), e1003926. 10.1371/journal.pcbi.1003926 (2014). 25474468 10.1371/journal.pcbi.1003926 PMC4256011

[55] Rigden, D. J. & Galperin, M. Y. Sequence analysis of GerM and SpoVS, uncharacterized bacterial ‘sporulation’ proteins with widespread phylogenetic distribution. *Bioinformatics* **24**(16), 1793–1797. 10.1093/bioinformatics/btn314 (2008). 18562273 10.1093/bioinformatics/btn314 PMC2732212

[56] Steinegger, M. et al. HH-suite3 for fast remote homology detection and deep protein annotation. *BMC Bioinform.* **20**(1), 1–15. 10.1186/S12859-019-3019-7/FIGURES/7 (2019). 10.1186/s12859-019-3019-7 PMC6744700 31521110

[57] Baek, M. et al. Accurate prediction of protein structures and interactions using a three-track neural network. *Science* **373**(6557), 871–876 (2021). 34282049 10.1126/science.abj8754 PMC7612213

[58] Zhang, C., Shine, M., Pyle, A. M. & Zhang, Y. US-align: Universal structure alignments of proteins, nucleic acids, and macromolecular complexes. *BioRxiv.*10.1101/2022.04.18.488565 (2022). 36038728 10.1038/s41592-022-01585-1

[59] Valdar, W. S. J. Scoring residue conservation. *Proteins* **48**(2), 227–241. 10.1002/prot.10146 (2002). 12112692 10.1002/prot.10146

[60] Perederina, A., Esakova, O., Koc, H., Schmitt, M. E. & Krasilnikov, A. S. Specific binding of a Pop6/Pop7 heterodimer to the P3 stem of the yeast RNase MRP and RNase P RNAs. *RNA* **13**(10), 1648–1655. 10.1261/rna.654407 (2007). 17717080 10.1261/rna.654407 PMC1986809

[61] Carroll, K., Gomez, C. & Shapiro, L. Tubby proteins: the plot thickens. *Nat Rev Mol Cell Biol.* **5**(1), 55–63. 10.1038/nrm1278 10.1038/nrm1278 14708010

[62] Alva, V., Söding, J. & Lupas, A. N. A vocabulary of ancient peptides at the origin of folded proteins. *eLife* **4**, e09410. 10.7554/eLife.09410 (2015). 26653858 10.7554/eLife.09410 PMC4739770

[63] Anantharaman, V., Koonin, E. V. & Aravind, L. Regulatory potential, phyletic distribution and evolution of ancient, intracellular small-molecule-binding domains1. *J. Mol. Biol.* **307**(5), 1271–1292. 10.1006/jmbi.2001.4508 (2001). 11292341 10.1006/jmbi.2001.4508

[64] Ponting, C. P. & Russell, R. R. The natural history of protein domains. *Annu. Rev. Biophys.* **31**, 45–71. 10.1146/annurev.biophys.31.082901.134314 (2002). 10.1146/annurev.biophys.31.082901.134314 11988462

[65] Alvarez-Carreño, C., Penev, P. I., Petrov, A. S. & Williams, L. D. Fold evolution before LUCA: Common ancestry of SH3 domains and OB domains. *Mol. Biol. Evol.* **38**(11), 5134–5143. 10.1093/molbev/msab240 (2021). 34383917 10.1093/molbev/msab240 PMC8557408

[66] Grau-Bové, X. et al. A phylogenetic and proteomic reconstruction of eukaryotic chromatin evolution. *Nat. Ecol. Evol.* **6**(7), 1007–1023. 10.1038/s41559-022-01771-6 (2022). 35680998 10.1038/s41559-022-01771-6 PMC7613034

[67] Longo, L. M. et al. On the emergence of P-Loop NTPase and Rossmann enzymes from a Beta-Alpha-Beta ancestral fragment. *eLife* **9**, e64415. 10.7554/eLife.64415 (2020). 33295875 10.7554/eLife.64415 PMC7758060

[68] Makarova, K. S. et al. Diversity, origin, and evolution of the ESCRT systems. *mBio* **15**(3), e0033524. 10.1128/mbio.00335-24 (2024). 38380930 10.1128/mbio.00335-24 PMC10936438

[69] Taujale, R. et al. Deep evolutionary analysis reveals the design principles of fold A glycosyltransferases. *eLife* **9**, e54532. 10.7554/eLife.54532 (2020). 32234211 10.7554/eLife.54532 PMC7185993

[70] Acharya, D. et al. Ectopic overexpression of *Plasmodium falciparum* DNA/RNA-binding Alba proteins misregulates virulence gene homeostasis during asexual blood development. *BioRxiv* **1**, 1 (2024). 10.1128/spectrum.00885-24 PMC11878077 39868986

[71] Diffendall, G. M. et al. Discovery of RUF6 ncRNA-interacting proteins involved in *P. falciparum* immune evasion. *Life Sci. Alliance* **6**(1), e202201577. 10.2508/lsa.202201577 (2023). 36379669 10.26508/lsa.202201577 PMC9670795

[72] Chamberlain, J. R., Lee, Y., Lane, W. S. & Engelke, D. R. Purification and characterization of the nuclear RNase P holoenzyme complex reveals extensive subunit overlap with RNase MRP. *Genes Dev.* **12**(11), 1678–1690. 10.1101/gad.12.11.1678 (1998). 9620854 10.1101/gad.12.11.1678 PMC316871

[73] Frank, D. N. & Pace, N. R. Ribonuclease P: Unity and diversity in a tRNA processing ribozyme. *Annu. Rev. Biochem.* **67**, 153–180. 10.1146/annurev.biochem.67.1.153 (1998). 9759486 10.1146/annurev.biochem.67.1.153

[74] Goldfarb, K. C. & Cech, T. R. Targeted CRISPR disruption reveals a role for RNase MRP RNA in human preribosomal RNA processing. *Genes Dev.* **31**(1), 59–71. 10.1101/gad.286963.116 (2017). 28115465 10.1101/gad.286963.116 PMC5287113

[75] Guerrier-Takada, C., Eder, P. S., Gopalan, V. & Altman, S. Purification and characterization of Rpp25, an RNA-binding protein subunit of human ribonuclease P. *RNA* **8**(3), 290–295. 10.1017/s1355838202027954 (2002). 12003489 10.1017/s1355838202027954 PMC1370251

[76] Jarrous, N. Roles of RNase P and its subunits. *Trends Genet.* **33**(9), 594–603. 10.1016/j.tig.2017.06.006 (2017). 28697848 10.1016/j.tig.2017.06.006

[77] Welting, T. J. M., Kikkert, B. J., van Venrooij, W. J. & Pruijn, G. J. M. Differential association of protein subunits with the human RNase MRP and RNase P complexes. *RNA* **12**(7), 1373–1382. 10.1261/rna.2293906 (2006). 16723659 10.1261/rna.2293906 PMC1484433

[78] Bhatta, A. & Hillen, H. S. Structural and mechanistic basis of RNA processing by protein-only ribonuclease P enzymes. *Trends Biochem Sci.* **47**(11), 965–977. 10.1016/j.tibs.2022.05.006 2022). 10.1016/j.tibs.2022.05.006 35725940

[79] Verma, J. K., Wardhan, V., Singh, D., Chakraborty, S. & Chakraborty, N. Genome-wide identification of the Alba gene family in plants and stress-responsive expression of the rice alba genes. *Genes* **9**(4), 183. 10.3390/genes9040183 (2018). 29597290 10.3390/genes9040183 PMC5924525

[80] Laursenm, S.P., Bowerman, S. & Luger K. A.The Final Frontier of Chromatin. *J Mol Biol. ***433**(6), 166791. 10.1016/j.jmb.2020.166791 (2020). 10.1016/j.jmb.2020.166791 PMC7987875 33383035

[81] Houser-Scott, F. et al. Interactions among the protein and RNA subunits of Saccharomyces cerevisiae nuclear RNase P. *Proc. Natl. Acad. Sci. U.S.A.* **99**(5), 2684–2689. 10.1073/pnas.052586299 (2002). 11880623 10.1073/pnas.052586299 PMC122408

[82] Koti, J., Kanugula, S. & Suryanarayana, T. DNA aggregation by an archaeal DNA binding protein Sac 10b and its novel DNA nicking activity. *Indian J. Biochem. Biophys.* **45**, 166–173 (2008).

[83] Tóth-Petróczy, A. & Tawfik, D. S. The robustness and innovability of protein folds. *Curr Opin Struct Biol.* 131–138 (2014). 10.1016/j.sbi.2014.06.007 25038399

[84] Chowdhury, M. N. & Jin, H. The RGG motif proteins: Interactions, functions, and regulations. *Wiley Interdiscip. Rev. RNA* **14**(1), e1748. 10.1002/wrna.1748 (2023). 35661420 10.1002/wrna.1748 PMC9718894

[85] Browning, D. F., Grainger, D. C. & Busby, S. J. Effects of nucleoid-associated proteins on bacterial chromosome structure and gene expression. *Curr Opin Microbiol. ***13**(6), 773–780. 10.1016/j.mib.2010.09.013 (2023). 10.1016/j.mib.2010.09.013 20951079

[86] Maruyama, H. et al. Histone and TK0471/TrmBL2 form a novel heterogeneousgenome architecture in the hyperthermophilic archaeon Thermococcus kodakarensis*Mol Biol Cell.* **23**(6), 386–398.10.1091/mbc.E10-08-0668 (2011). 10.1091/mbc.e10-08-0668 PMC3031468 21148291

[87] Li, L. et al. Wing phosphorylation is a major functional determinant of the Lrs14-type biofilm and motility regulator AbfR1 in Sulfolobus acidocaldarius.*Mol Microbiol.* **105**(5), 777–793.10.1111/mmi.13735 (2017). 10.1111/mmi.13735 28628237

[88] Napoli, A., Kvaratskelia, M., White, M. F., Rossi, M. & Ciaramella, M. A novel member of the bacterial-archaeal regulator family is a nonspecific dna-binding protein and induces positive supercoiling.*J Biol Chem.* **276**(14), 10745–10752. 10.1074/jbc.M010611200 (2001). 10.1074/jbc.M010611200 11148211

[89] Orell, A. et al. Lrs14 transcriptional regulators influence biofilm formation andcell motility of Crenarchaea. * ISME J.* **10**, 1886–1898. 10.1038/ismej.2013.68 (2013). 10.1038/ismej.2013.68 PMC3965304 23657363

[90] Söding, J., Biegert, A. & Lupas, A. N. The HHpred interactive server for protein homology detection and structure prediction. *Nucleic Acids Res.* **33**, W244–W248. 10.1093/nar/gki408 (2005). 15980461 10.1093/nar/gki408 PMC1160169

[91] Ondov, B. D., Bergman, N. H. & Phillippy, A. M. Interactive metagenomic visualization in a Web browser. *BMC Bioinform.* **12**, 385. 10.1186/1471-2105-12-385 (2011). 10.1186/1471-2105-12-385 PMC3190407 21961884

[92] Mirdita, M. et al. Uniclust databases of clustered and deeply annotated protein sequences and alignments. *Nucleic Acids Res.* **45**(D1), D170–D176. 10.1093/nar/gkw1081 (2017). 27899574 10.1093/nar/gkw1081 PMC5614098

[93] Rehse, P. H. & Yokoyama, S. *Stage V Sporolation Protein S (SPOVS) from Thermus thermophilus Zinc Form* (2007).

[94] Ostheimer, G. J., Barkan, A. & Matthews, B. W. Crystal structure of *E. coli* YhbY: A representative of a novel class of RNA binding proteins. *Structure* **10**(11), 1593–1601. 10.1016/s0969-2126(02)00886-9 (2002). 12429100 10.1016/s0969-2126(02)00886-9

[95] De Cock, E., Garcia, C. & Dardel, F. *Translation Initiation Factor if3 from Escherichia coli Ribosome Binding Domain* (1998).

[96] Schrödinger, LLC. *The PyMOL Molecular Graphics System, Version 2.5.2* (2015).

[97] Baker, N. A., Sept, D., Joseph, S., Holst, M. J. & McCammon, J. A. Electrostatics of nanosystems: Application to microtubules and the ribosome. *Proc. Natl. Acad. Sci.* **98**(18), 10037–10041. 10.1073/pnas.181342398 (2001). 11517324 10.1073/pnas.181342398 PMC56910

[98] Katoh, K., Rozewicki, J. & Yamada, K. D. MAFFT online service: Multiple sequence alignment, interactive sequence choice and visualization. *Brief. Bioinform.* **20**(4), 1160–1166. 10.1093/bib/bbx108 (2019). 28968734 10.1093/bib/bbx108 PMC6781576

[99] Kumarevel, T. et al. Crystal structure of an archaeal specific DNA-binding protein (Ape10b2) from *Aeropyrum pernix* K1. *Proteins Struct. Funct. Genet.* **71**(3), 1156–1162. 10.1002/prot.21807 (2008). 18004791 10.1002/prot.21807

[100] Zhao, K., Chai, X. & Marmorstein, R. Structure of a Sir2 substrate, Alba, reveals a mechanism for deacetylation-induced enhancement of DNA binding. *J. Biol. Chem.* **278**(28), 26071–26077. 10.1074/jbc.M303666200 (2003). 12730210 10.1074/jbc.M303666200

[101] Fr, F. et al. Gotree/Goalign: Toolkit and Go API to facilitate the development of phylogenetic workflows. *NAR Genom. Bioinform.* **3**(3), 75. 10.1093/NARGAB/LQAB075 (2021). 10.1093/nargab/lqab075 PMC8356961 34396097

[102] Minh, B. Q. et al. IQ-TREE 2: New models and efficient methods for phylogenetic inference in the genomic era. *Mol. Biol. Evol.* **37**(5), 1530–1534. 10.1093/MOLBEV/MSAA015 (2020). 32011700 10.1093/molbev/msaa015 PMC7182206

[103] Kalyaanamoorthy, S., Minh, B. Q., Wong, T. K. F., Von Haeseler, A. & Jermiin, L. S. ModelFinder: Fast model selection for accurate phylogenetic estimates. *Nat. Methods* **14**(6), 587–589. 10.1038/nmeth.4285 (2017). 28481363 10.1038/nmeth.4285 PMC5453245

[104] Letunic, I. & Bork, P. Interactive tree of life (iTOL) v5: An online tool for phylogenetic tree display and annotation. *Nucleic Acids Res.* **49**(W1), W293–W296. 10.1093/nar/gkab301 (2021). 33885785 10.1093/nar/gkab301 PMC8265157

[105] Tamura, K. et al. Estimating divergence times in large molecular phylogenies. *Proc. Natl. Acad. Sci.* **109**(47), 19333–19338. 10.1073/pnas.1213199109 (2012). 23129628 10.1073/pnas.1213199109 PMC3511068

[106] Tamura, K., Tao, Q. & Kumar, S. Theoretical foundation of the RelTime method for estimating divergence times from variable evolutionary rates. *Mol. Biol. Evol.* **35**(7), 1770–1782. 10.1093/molbev/msy044 (2018). 29893954 10.1093/molbev/msy044 PMC5995221

[107] Tamura, K., Stecher, G. & Kumar, S. MEGA11: Molecular evolutionary genetics analysis version 11. *Mol. Biol. Evol.* **38**(7), 3022–3027. 10.1093/molbev/msab120 (2021). 33892491 10.1093/molbev/msab120 PMC8233496

[108] Kumar, S. et al. TimeTree 5: An expanded resource for species divergence times. *Mol. Biol. Evol.* **39**(8), 174. 10.1093/molbev/msac174 (2022). 10.1093/molbev/msac174 PMC9400175 35932227

