# Supplementary material for: Evolution of sequence, structural and functional diversity of the ubiquitous DNA/RNA-binding Alba domain
Source: Sci Rep. 2024 Dec 5;14:30363. doi: 10.1038/s41598-024-79937-4 (PMC11621453; doi:10.1038/s41598-024-79937-4)
Supplement: Supplementary file 1 — Supplementary Information. [file 41598_2024_79937_MOESM1_ESM.pdf]

Supplementary Information for Jagadeesh and Vembar, 2024

This document contains:

Supplementary Tables S1 and S2

Supplementary Figures S1 to S8

Provided as links:

Shiny R app summarising the Alba-domain proteins identified in this study with their NCBI accession ID and the SSN cluster that they belong to

http://plasmobees.shinyapps.io/Alba_domain_containing_proteins

Phylogenetic tree shown in Figure 5

https://itol.embl.de/tree/4947193194102417268539 14

Supplementary Table S1: Summary of co-occurring domains in Alba domain-containing proteins. Domains that co-occur in a minimum of five Alba hits are listed below.

Domain Name(s)	Domain Function(s)	InterPro/PFAM ID(s)
Thioredoxin-like_fold	Regulates the activity of target proteins by changing the redox state of thiol groups (S2 to SH2)	IPR012336
TAXi_N; TAXi_C	Aspartic proteinase and xylanase inhibitory activity	IPR032861; IPR032799
HHH_5	Helix-hairpin-helix DNA-binding domain	PF14520
TULP	Tubby-like domain thought to have transcription factor activity	IPR000007
PCI_dom; 26S_Psome_reg_C	Proteasome, COP9, Initiation factor 3 or PCI domain stabilizes protein-protein interactions and co-occurs with the 26S_Psome_reg_C domain, which is found at the C-terminus of 26S proteasome regulatory subunits.	IPR000717; IPR013586
DCTN3	Dynactin subunit 3, which may play a role in cytokinesis	IPR009991
LMBR1-like_membr_prot	LMBR1-like membrane protein, which may play a role in cell signaling	IPR006876
PFD_beta-like	Prefoldin beta-like; protein chaperone	IPR002777
PsdUridine_synth_N	Pseudouridine synthase II, N-terminal; catalyse the isomerisation of uridine to pseudouridine (Psi) in a variety of RNA molecules.	IPR002501
KH_dom_type_1	K Homology domain, type 1 is a RNA-binding domain found in a variety of proteins that regulate eseential processes such as splicing, etc.	IPR004088
Cyt_c_oxidase_su5A/6	Subunits Va and VI are the heme A-containing chains of cytochrome c oxidase. Subunit Va is located on the matrix side of the membrane and binds thyroid hormone T2, releasing allosteric inhibition caused by the binding of ATP to subunit IV and allowing high turnover at elevated intramitochondrial ATP/ADP ratios.	IPR003204
5'(3')-deoxyribonucleotidase	Not known	IPR010708

Supplementary Table S2: Summary of the 13 SSN clusters identified in this study and the number of Alba domain-containing proteins belonging to each cluster. The interactive Krona charts are deposited in https://github.com/jaiganeshjg/Alba as html files and can be accessed by clicking the provided hyperlink.

Cluster	Number of sequences	Krona chart
Alba domain-containing proteins	15161	https://raw.githack.com/jaiganeshjg/Alba/main/All_Alba.tsv.krona.html
Rpp25	5095	https://raw.githack.com/jaiganeshjg/Alba/main/Rpp25.tsv.krona.html
Archaeal Alba	3913	https://raw.githack.com/jaiganeshjg/Alba/main/ArchaealAlba.tsv.krona.html
Plant Alba	1781	https://raw.githack.com/jaiganeshjg/Alba/main/PlantAlba%E2%80%93At2g34160like.tsv.krona.html
Rpp20	1460	https://raw.githack.com/jaiganeshjg/Alba/main/Rpp20.tsv.krona.html
Pop7	1034	https://raw.githack.com/jaiganeshjg/Alba/main/Pop7.tsv.krona.html
Fungi alba3	676	https://raw.githack.com/jaiganeshjg/Alba/main/FungiAlba3.tsv.krona.html
Fungi alba2	433	https://raw.githack.com/jaiganeshjg/Alba/main/FungiAlba2.tsv.krona.html
Fungi alba1	360	https://raw.githack.com/jaiganeshjg/Alba/main/FungiAlba1.tsv.krona.html
PfAlba3 and PfAlba4	152	https://raw.githack.com/jaiganeshjg/Alba/main/PfAlba3_and_PfAlba4.tsv.krona.html
Pop6	145	https://raw.githack.com/jaiganeshjg/Alba/main/Pop6.tsv.krona.html
PfAlba5	53	https://raw.githack.com/jaiganeshjg/Alba/main/PfAlba5.tsv.krona.html
PfAlba6	39	https://raw.githack.com/jaiganeshjg/Alba/main/PfAlba6.tsv.krona.html
ThAlba	20	https://raw.githack.com/jaiganeshjg/Alba/main/ThAlba.tsv.krona.html

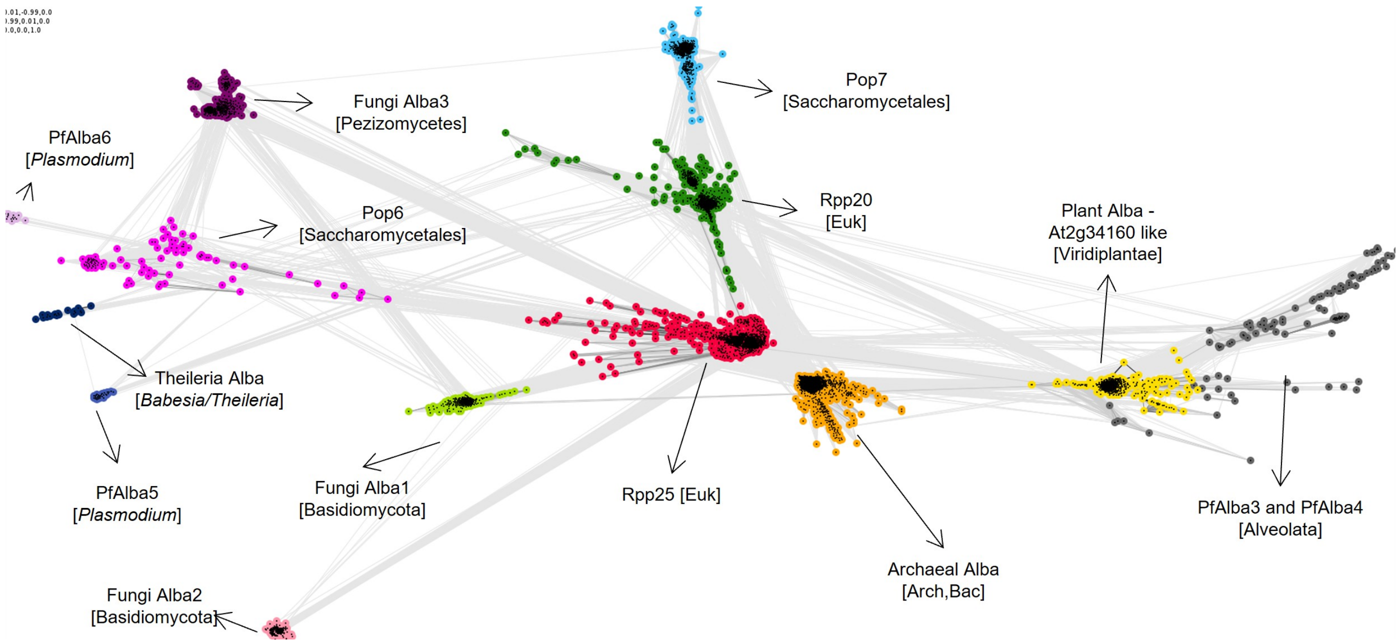

Supplementary Figure S1. CLANS Cluster map of a representative set of 8390 Alba homologs obtained after clustering the 15161 Albas identified in this study at a maximum pairwise identity of 90% using MMseqs2 (Steinegger et al., 2021). Dots represent sequences which were clustered at a p-value cutoff of 1e-4. Sequences belonging to the same cluster are similarly coloured. The degree of their pairwise sequence similarities, as measured by BLAST+ p-values, is reflected in line colouring; the darker a line, the greater the similarity.

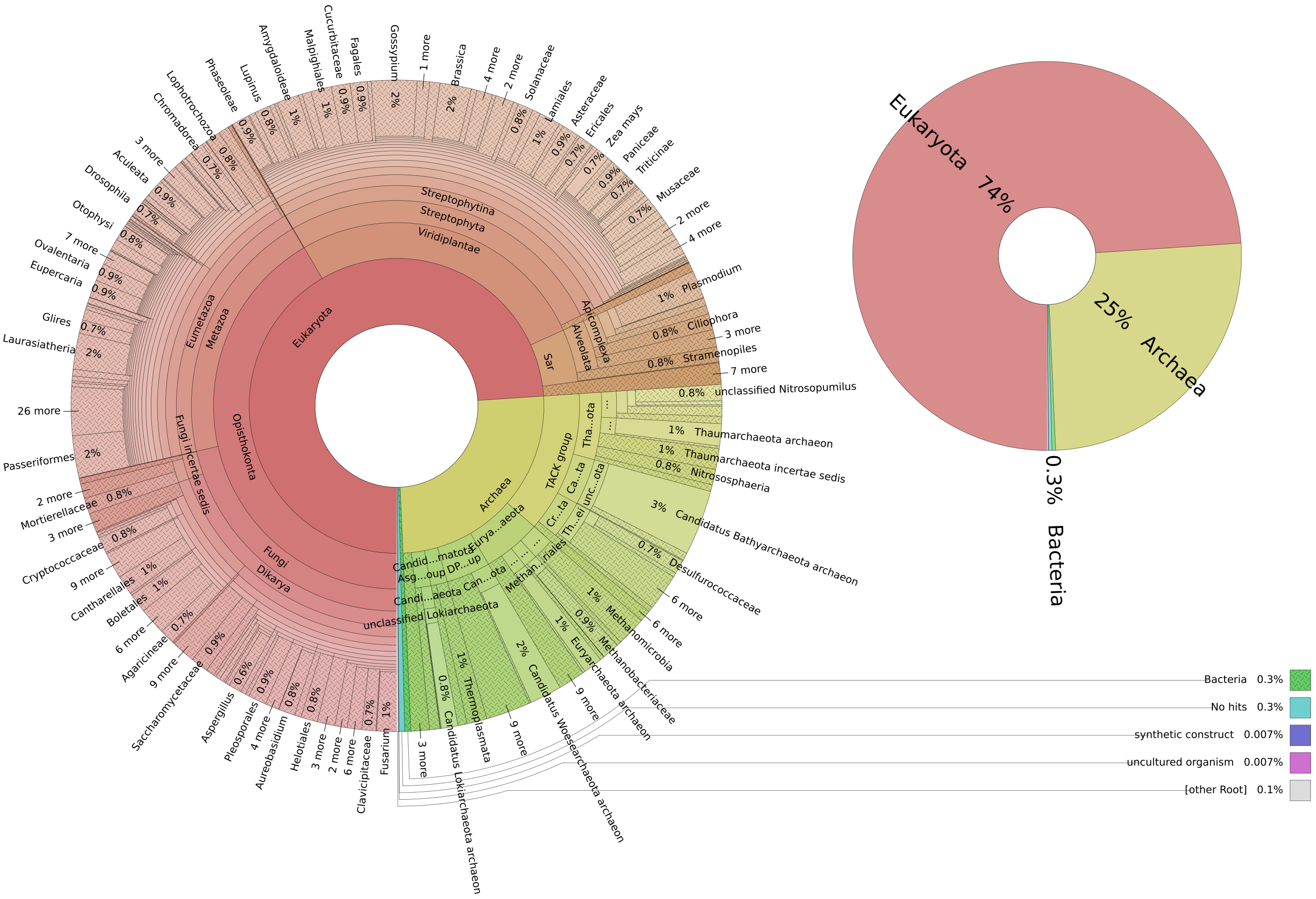

Supplementary Figure S2. Taxonomic distribution of 15161 Alba domain-containing proteins. ***Left:*** A pie chart was generated in Krona (Ondov et al., 2011) to represent the taxonomic distribution of all 15161 Alba domain-containing proteins identified in this study. The innermost rings represent domain and kingdom level classification. Proceeding outwards, the taxonomic resolution increases, *i.e.,* Phylum, Class, Order, Family, Genus, and Species being the outermost ring. ***Right***: A Krona pie-chart showing domain-level taxonomic distribution of the 15161 Alba homologs. The Krona charts are available as interactive html format. Refer to Supplementary Table S1 for more details.

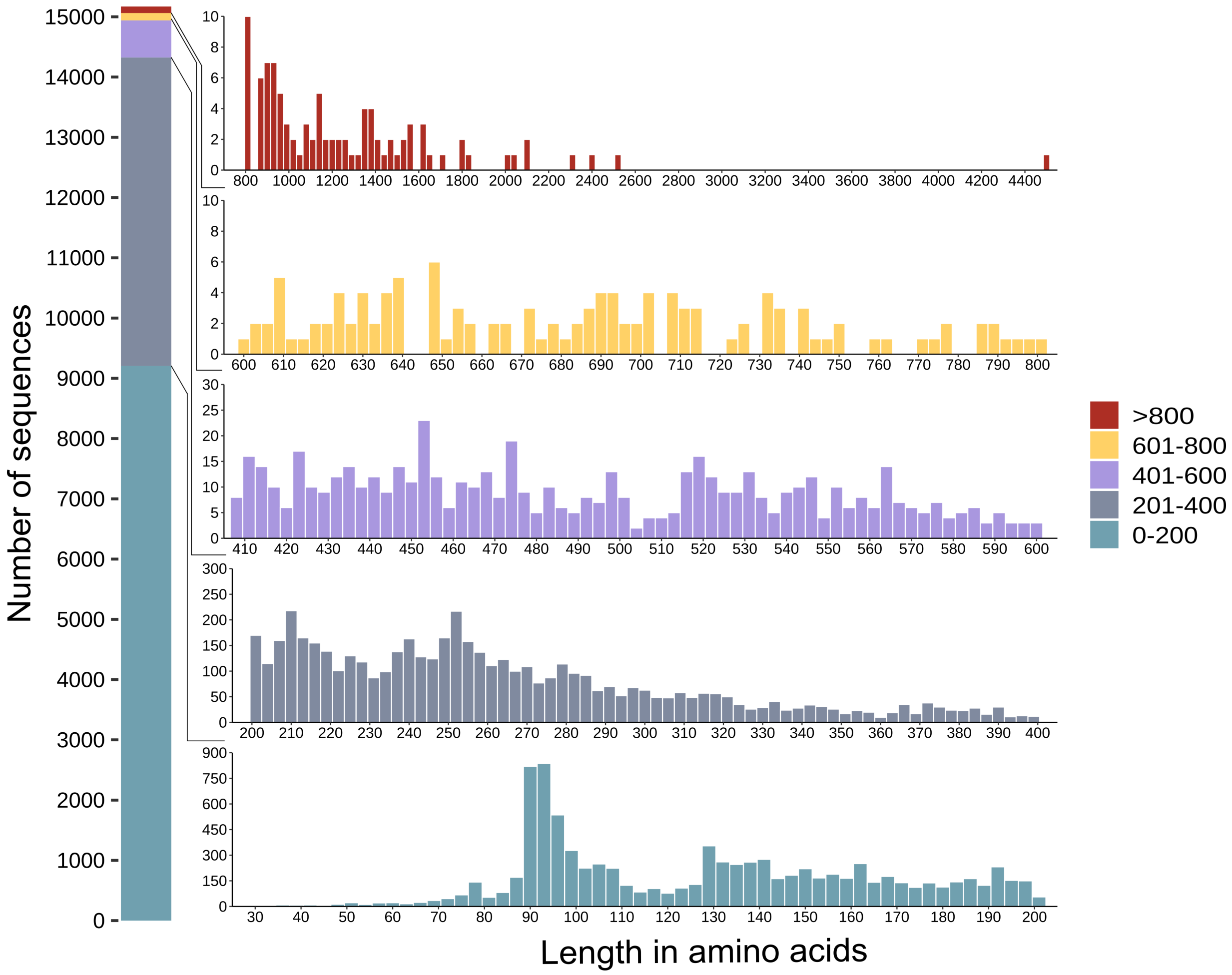

Supplementary Figure S3. Amino acid sequence length distribution of 15161 Alba-domain containing proteins. The X-axis corresponds to the length of the protein in amino acids, and the Y-axis to the number of hits for a given length.

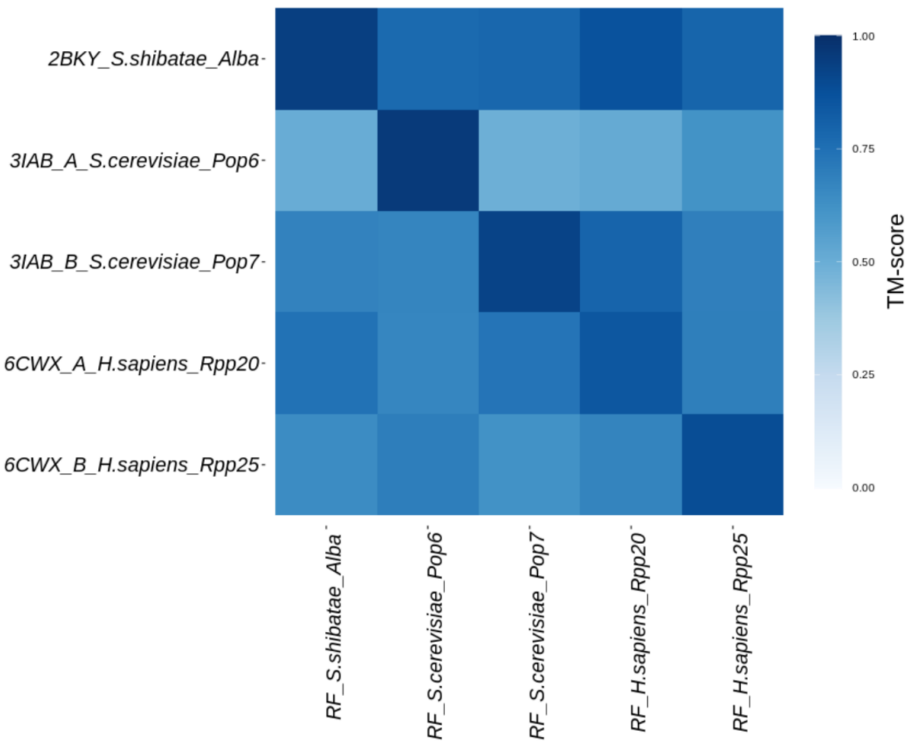

Supplementary Figure S4. Quantitative pairwise comparison of the solved crystal structures and RoseTTAFold-predicted structures of select Alba domains. TM-align (Zhang et al., 2022) was used to compare the crystal structures (y-axis) of five Albas to their *ab initio* structures (x-axis) predicted using RoseTTAFold (Baek et al., 2021). Crystal structures were downloaded from the RCSB-PDB database and their PDB ID is provided. RF = RosettaFold. The scale of the heatmap shows the TM-score reported by TM-align.

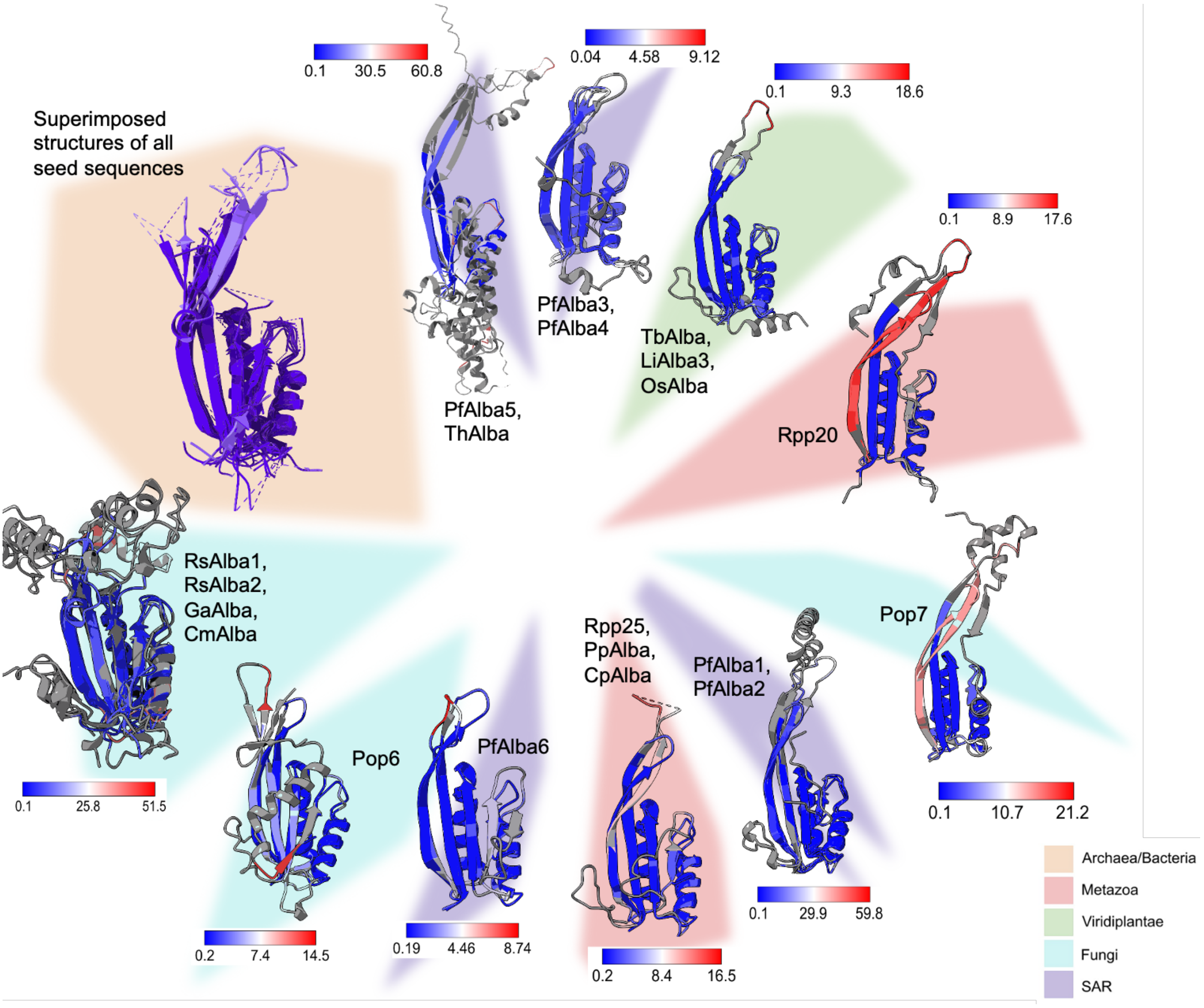

Supplementary Figure S5. Structural deviations of Alba seed sequence models. RosettaFold-predicted structures of Alba seed sequences (shown in Figure 3) from each clade of the phylogenetic tree were overlaid onto the crystal structure of *Sulfolobus solfataricus* Alba (SsAlba, PDB ID: 2BKY ((Jelinska et al., 2005)) using ChimeraX Matchmaker (Pettersen et al., 2004). Residues absent in the alignment are shown in grey. The scale bar depicts root mean square deviation or RMSD values calculated by ChimeraX, with blue indicating minimal deviation and red representing maximum deviation.

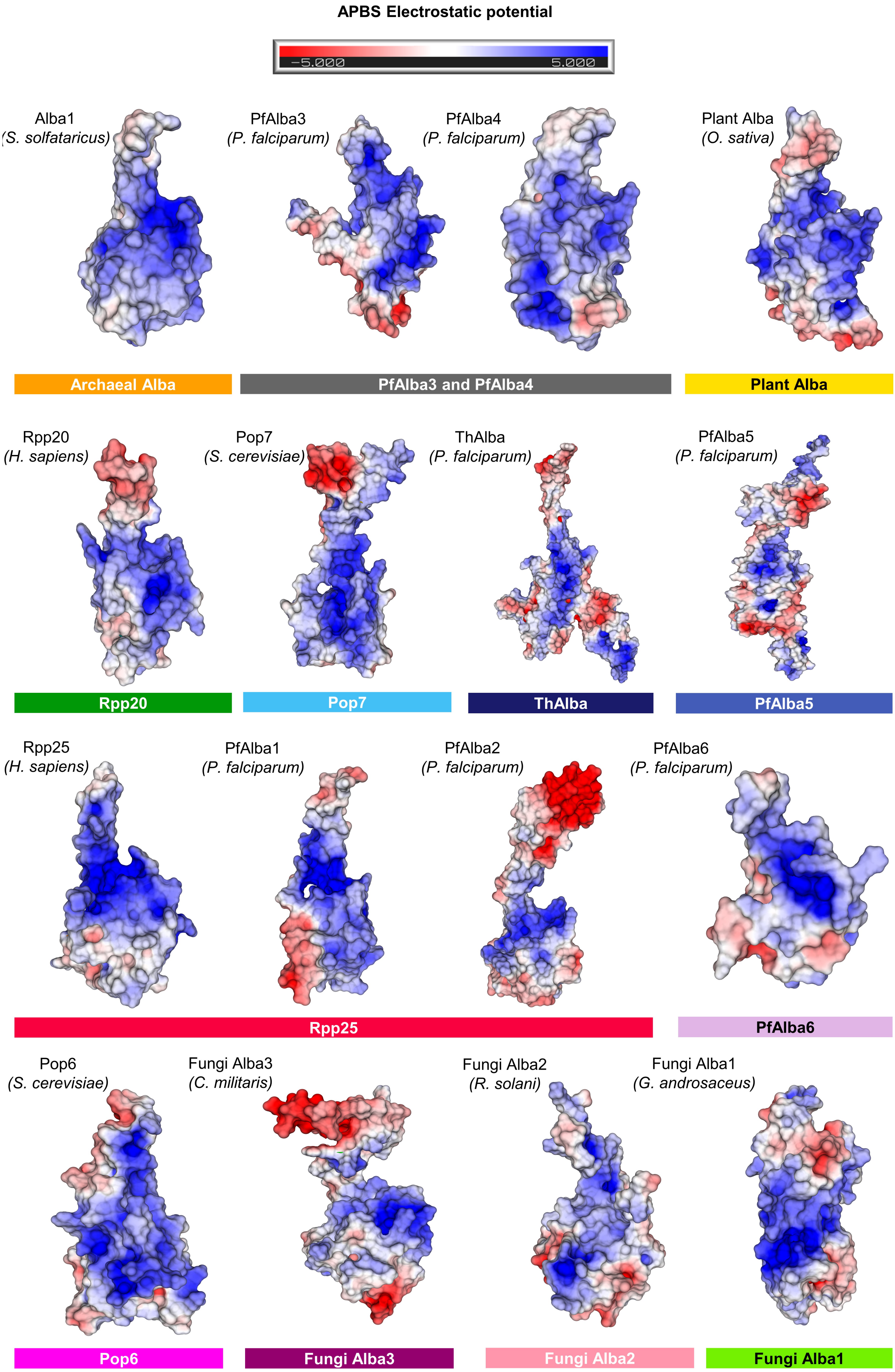
APBS Electrostatic potential
-5.000
5.000
Alba1
(S. solfataricus)
PfAlba3
(P. falciparum)
PfAlba4
(P. falciparum)
Plant Alba
(O. sativa)
Archaeal Alba
PfAlba3 and PfAlba4
Plant Alba
Rpp20
(H. sapiens)
Pop7
(S. cerevisiae)
ThAlba
(P. falciparum)
PfAlba5
(P. falciparum)
Rpp20
Pop7
ThAlba
PfAlba5
Rpp25
(H. sapiens)
PfAlba1
(P. falciparum)
PfAlba2
(P. falciparum)
PfAlba6
(P. falciparum)
Rpp25
PfAlba6
Pop6
(S. cerevisiae)
Fungi Alba3
(C. militaris)
Fungi Alba2
(R. solani)
Fungi Alba1
(G. androsaceus)
Pop6
Fungi Alba3
Fungi Alba2
Fungi Alba1

Supplementary Figure S6. The DNA/RNA-interacting electropositive surface is conserved across all Alba homologs. For one representative structure from each cluster, as well as for the predicted structures of all six *Plasmodium* Albas, Adaptive Poisson-Boltzmann Solver (APBS; (Baker et al., 2001)) surface maps were generated and rendered at +/- 5 kT/e. The scale bar represents charge, with blue indicating electropositivity and red indicating electronegativity.

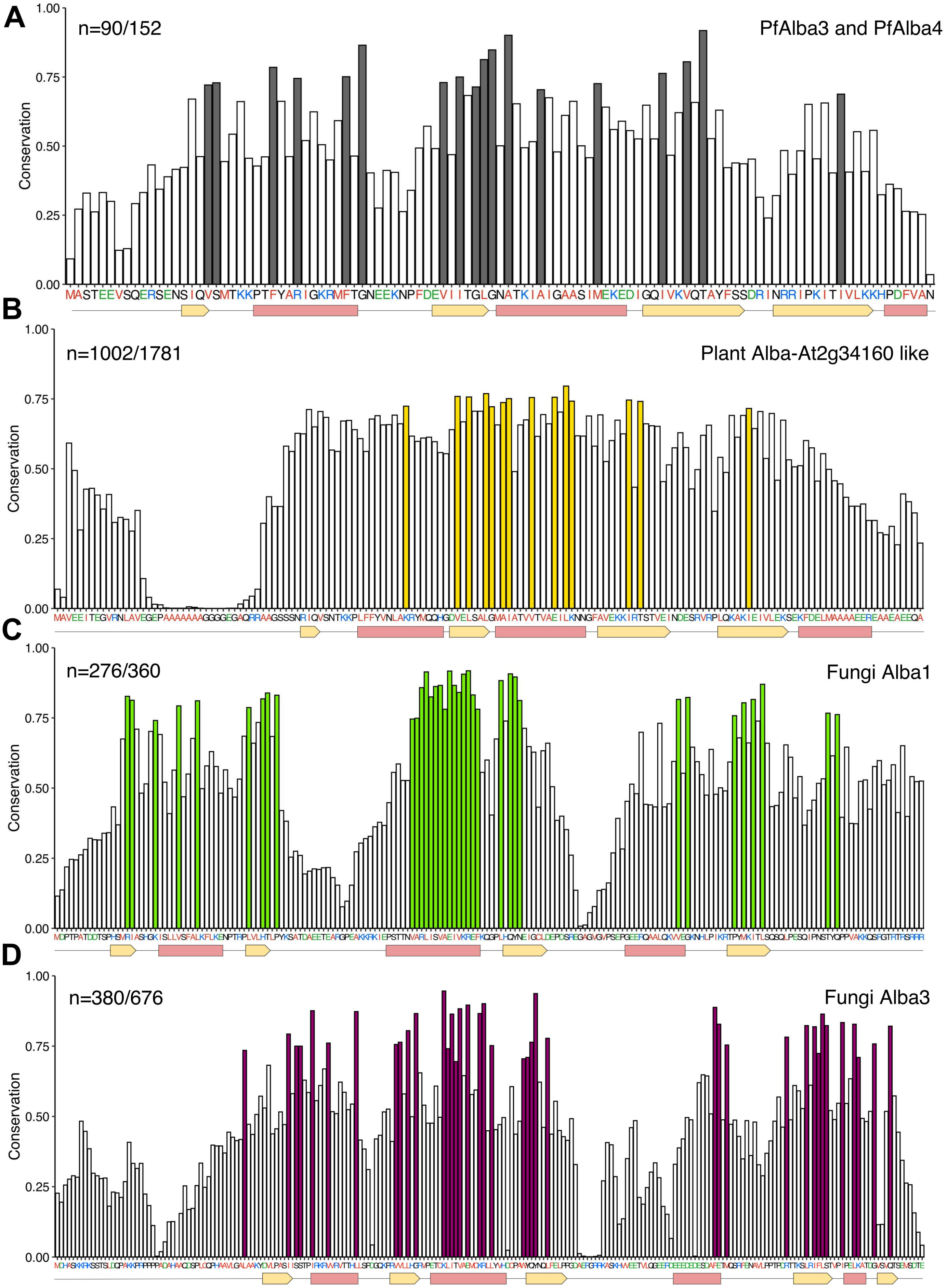

A
n=90/152
PfAlba3 and PfAlba4
B
n=1002/1781
Plant Alba-At2g34160 like
C
n=276/360
Fungi Alba1
D
n=380/676
Fungi Alba3
Conservation
1.00
0.75
0.50
0.25
0.00
MASTEEVSQERSENSIQVSMTKKPTFYARIGKRMFTGNEEKNPFDEVIITGLGNATKIAIGAASIMEKEDIGQIVKVQTAYFSSDRINRRIPKITIVLKKHPDFVAN

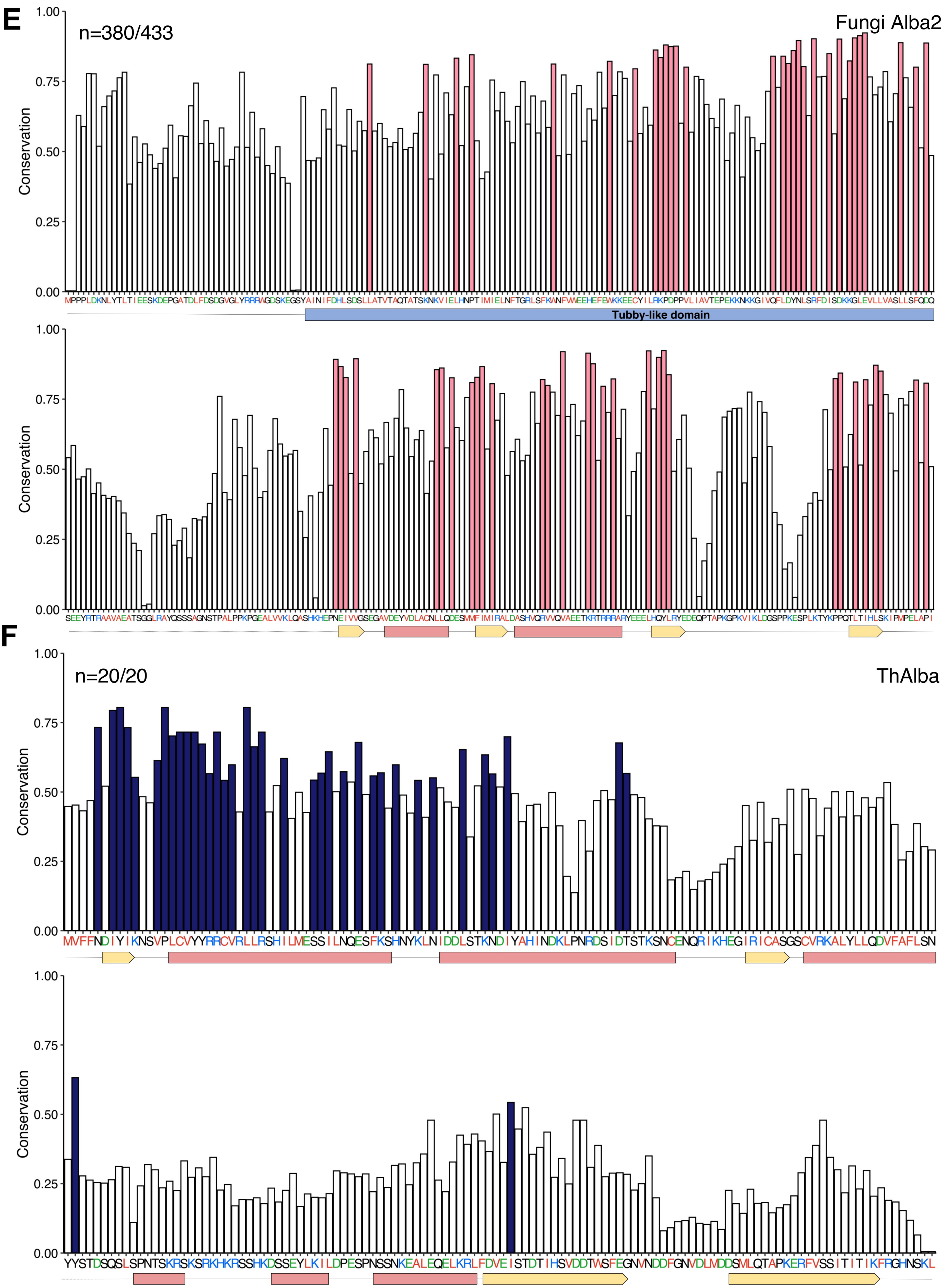
E
n=380/433
Fungi Alba2
Conservation
1.00
0.75
0.50
0.25
0.00
Tubby-like domain
F
n=20/20
ThAlba

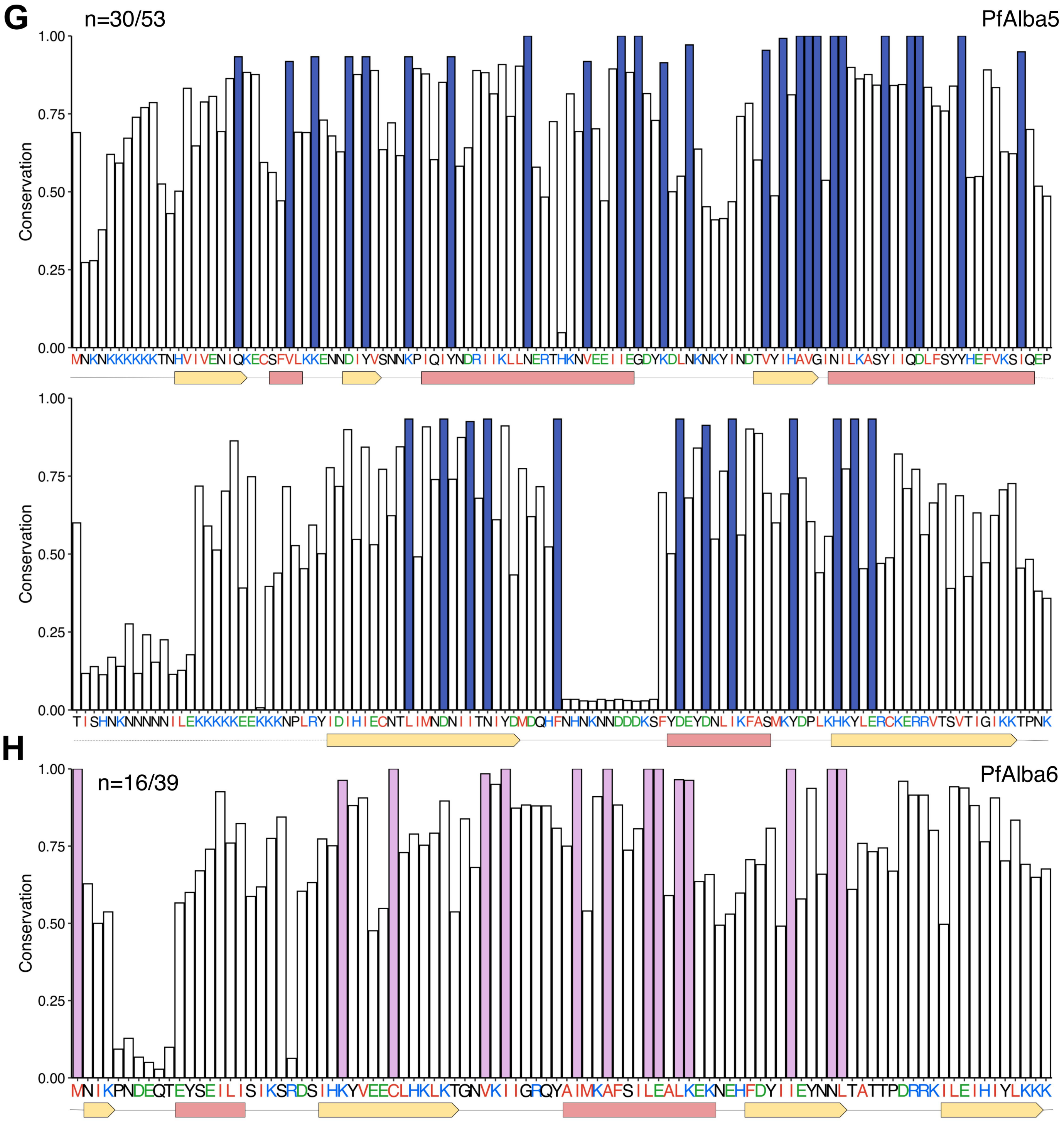

Supplementary Figure S7. Sequence Conservation of Alba proteins from eight different SSN clusters. The conservation (Y-axis) of the indicated number of proteins from, **A.** PfAlba3/PfAlba4-like cluster; **B.** Plant Alba-like cluster**; C.** Fungi Alba1 cluster; **D.** Fungi Alba3 cluster; **E.** Fungi Alba2 cluster; **F.** Alba-like proteins from *Babesia* and *Thileria* species; **G.** *Plasmodium* Alba5 cluster; or **H.** *Plasmodium* Alba6 cluster, was scored against the 'seed' sequence of that particular cluster using the ScoreCons server (Valdar, 2002). The number of aligned sequences out of the total number of sequences is shown. Conserved residues of at least one standard deviation more than the mean conservation across the alignment are shown as colour-filled bars, and their colour corresponds to the cluster colour in Fig. 1B. In the consensus sequence (X-axis), red represents hydrophobic residues, blue represents positively charged residues, and green represents negatively charged residues. The protein secondary structure is plotted along the X-axis with α-helices coloured in pink and β-sheets in yellow.

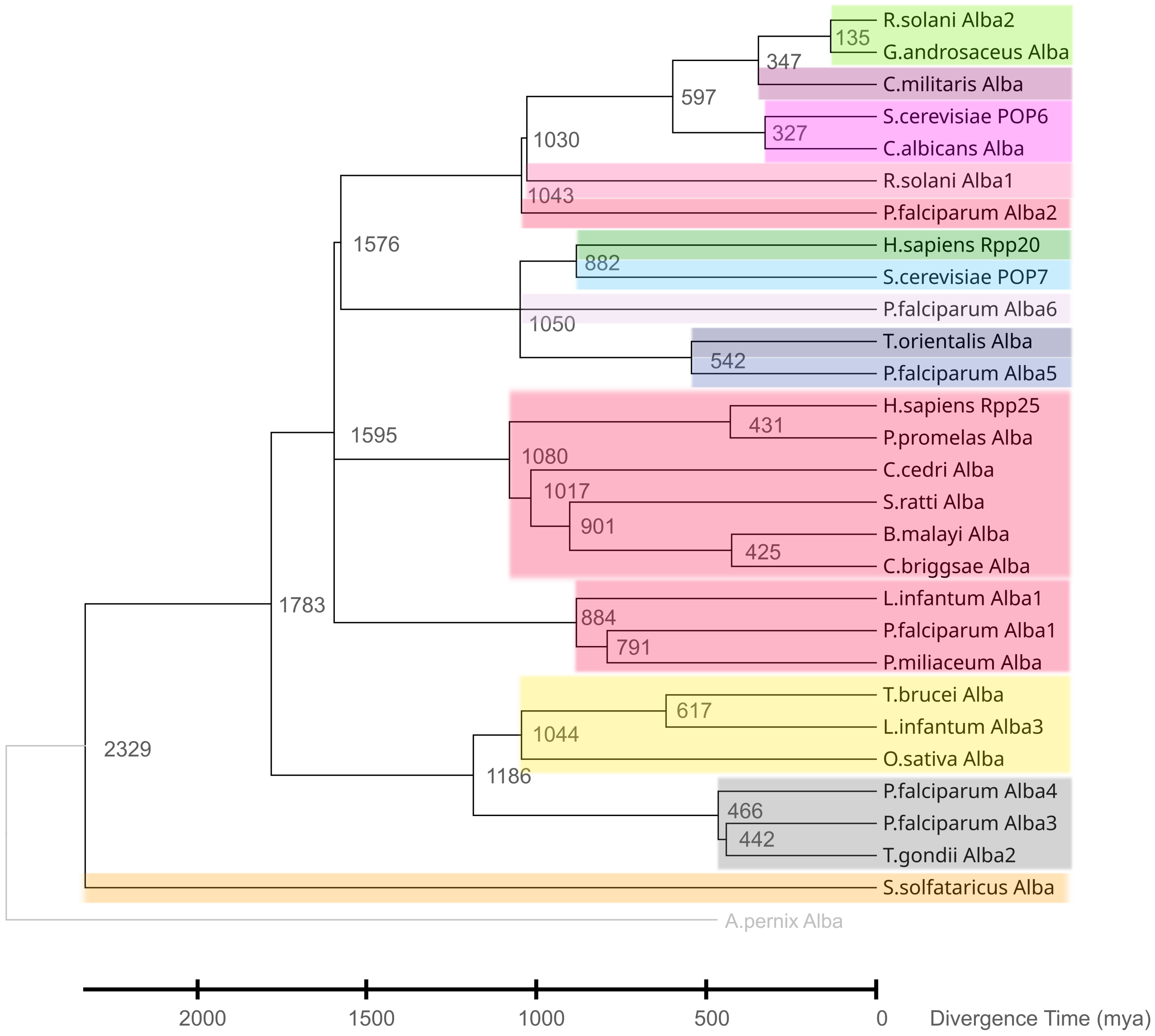

Supplementary Figure S8. Time-divergence phylogenetic tree of seed Alba sequences. A timetree inferred by applying the RelTime (Tamura et al., 2012, 2018) method to the Maximum-Likelihood phylogenetic tree of 29 'seed' Alba sequences (excluding bacterial Albas) was generated using Iqtree2 (Minh et al., 2020). The timetree was computed using three calibration constraints: *H. sapiens* Rpp25-*P. promelas* Alba, *C. albicans* Alba-*R. solani* Alba2, and *T. brucei* Alba-*L. infantum* Alba3. The Tao et al. method (Tao et al., 2020) was used to set minimum and maximum time boundaries on nodes for which calibration densities were provided from TimeTree 5 database (Kumar et al., 2022). Confidence intervals were computed using the Tao et al. method.

List of References

1. Baek, M., DiMaio, F., Anishchenko, I., Dauparas, J., Ovchinnikov, S., Lee, G. R., Wang, J., Cong, Q., Kinch, L. N., Dustin Schaeffer, R., Millán, C., Park, H., Adams, C., Glassman, C. R., DeGiovanni, A., Pereira, J. H., Rodrigues, A. V., Van Dijk, A. A., Ebrecht, A. C., … Baker, D. (2021). Accurate prediction of protein structures and interactions using a three-track neural network. *Science*, *373*(6557), 871–876. https://doi.org/10.1126/SCIENCE.ABJ8754/SUPPL_FILE/ABJ8754_MDAR_REPRODUCIBILITY_CHECKLIST.PDF
2. Baker, N. A., Sept, D., Joseph, S., Holst, M. J., & McCammon, J. A. (2001). Electrostatics of nanosystems: Application to microtubules and the ribosome. *Proceedings of the National Academy of Sciences*, *98*(18), 10037–10041. https://doi.org/10.1073/pnas.181342398
3. Jelinska, C., Conroy, M. J., Craven, C. J., Hounslow, A. M., Bullough, P. A., Waltho, J. P., Taylor, G. L., & White, M. F. (2005). Obligate heterodimerization of the archaeal Alba2 protein with Alba1 provides a mechanism for control of DNA Packaging. *Structure*, *13*(7), 963–971. https://doi.org/10.1016/j.str.2005.04.016
4. Kumar, S., Suleski, M., Craig, J. M., Kasprowicz, A. E., Sanderford, M., Li, M., Stecher, G., & Hedges, S. B. (2022). TimeTree 5: An Expanded Resource for Species Divergence Times. *Molecular Biology and Evolution*, *39*(8), msac174. https://doi.org/10.1093/molbev/msac174
5. Minh, B. Q., Schmidt, H. A., Chernomor, O., Schrempf, D., Woodhams, M. D., Von Haeseler, A., Lanfear, R., & Teeling, E. (2020). IQ-TREE 2: New Models and Efficient Methods for Phylogenetic Inference in the Genomic Era. *Molecular Biology and Evolution*, *37*(5), 1530–1534. https://doi.org/10.1093/MOLBEV/MSAA015
6. Ondov, B. D., Bergman, N. H., & Phillippy, A. M. (2011). Interactive metagenomic visualization in a Web browser. *BMC Bioinformatics*, *12*. https://doi.org/10.1186/1471-2105-12-385
7. Pettersen, E. F., Goddard, T. D., Huang, C. C., Couch, G. S., Greenblatt, D. M., Meng, E. C., & Ferrin, T. E. (2004). UCSF Chimera—A visualization system for exploratory research and analysis. *Journal of Computational Chemistry*, *25*(13), 1605–1612. https://doi.org/10.1002/JCC.20084
8. Steinegger, M., Mirdita, M., Karin, E. L., Von Den Driesch, L., Galiez, C., & Söding, J. (2021). *MMseqs2 User Guide*.
9. Tamura, K., Battistuzzi, F. U., Billing-Ross, P., Murillo, O., Filipski, A., & Kumar, S. (2012). Estimating divergence times in large molecular phylogenies. *Proceedings of the National Academy of Sciences*, *109*(47), 19333–19338. https://doi.org/10.1073/pnas.1213199109
10. Tamura, K., Tao, Q., & Kumar, S. (2018). Theoretical Foundation of the RelTime Method for Estimating Divergence Times from Variable Evolutionary Rates. *Molecular Biology and Evolution*, *35*(7), 1770–1782. https://doi.org/10.1093/molbev/msy044
11. Tao, Q., Tamura, K., Mello, B., & Kumar, S. (2020). Reliable Confidence Intervals for RelTime Estimates of Evolutionary Divergence Times. *Molecular Biology and Evolution*, *37*(1), 280–290. https://doi.org/10.1093/molbev/msz236
12. Valdar, W. S. J. (2002). Scoring residue conservation. *Proteins*, *48*(2), 227–241. https://doi.org/10.1002/prot.10146
13. Zhang, C., Shine, M., Pyle, A. M., & Zhang, Y. (2022). US-align: Universal structure alignments of proteins, nucleic acids, and macromolecular complexes. *Nature Methods*, *19*(9), 1109–1115. https://doi.org/10.1038/s41592-022-01585-1
